# Unraveling the development of cutaneous neurofibromas in neurofibromatosis type 1

**DOI:** 10.1186/s40478-025-02075-z

**Published:** 2025-07-19

**Authors:** Pernelle Pulh, Fanny Coulpier, Audrey Onfroy, Layna Oubrou, Wanzhen Zhang, Léa Toledano, Elie Abou Zougheib, Laura Fertitta, Pierre Wolkenstein, Piotr Topilko

**Affiliations:** 1 Institut Mondor de Recherche Biomédicale, Inserm U955 Team 9, 8 rue du Général Sarrail, Créteil, 94000 France; 2 Department of Dermatology, GHU Henri-Mondor hospital, AP-HP, 1 Rue Gustave Eiffel, Créteil, 94000 France; 3 Paris-Est Créteil University, 61 Av. du Général de Gaulle, Créteil, 94000 France

**Keywords:** Neurofibromatosis type 1, Cutaneous neurofibromas, Schwann cells, Fibroblasts, Fibrosis, Skin innervation

## Abstract

Neurofibromatosis type 1 (NF1) is a genetic disorder that leads to the formation of cutaneous neurofibromas (cNFs), benign nerve sheath tumors that develop in the skin and significantly impact the quality of life of patients. cNF development begins with bi-allelic NF1 loss in the Schwann cell (SC) lineage, followed by the recruitment of a complex tumor microenvironment consisting of fibroblasts, immune cells, blood vessels, axons, and a dense extracellular matrix. Despite its high prevalence and clinical impact, the molecular mechanisms underlying cNF formation remain poorly understood. Here, we used an *Nf1* knockout (*Nf1-KO*) mouse model combined with immunohistochemistry and single cell transcriptomics in order to investigate the mechanisms driving cNF development. Our results showed that mutant SCs accumulate in the skin of young mice weeks prior to the onset of cNF. However, these cells remain quiescent until triggered by skin trauma, which induces their proliferation and the rapid formation of cNFs. Using a trauma-induced *Nf1*-KO model with scRNAseq, we designed a transcriptomic atlas of growing and mature cNFs, as well as adjacent apparently healthy skin. This analysis identified a population of non-myelinating Aquaporin1^high^Nestin^low^ SCs as the likely cells of origin for cNFs. These cells overexpress genes involved in axon growth and guidance, potentially driving the abnormal innervation observed in both mouse and patient cNFs. In addition, we found that tumor SCs, along with dermal and/or epineurial fibroblasts and pericytes, overexpress genes encoding collagen, contributing to the extensive fibrosis characteristic of cNFs. Notably, all of these cells exhibit high expression of periostin and tenascin C, key extracellular matrix components, highlighting them as novel therapeutic targets in view of cNF treatment.

**Supplementary Information:**

The online version contains supplementary material available at 10.1186/s40478-025-02075-z.

## Introduction

Neurofibromatosis type 1 (NF1) is an autosomal dominant genetic disorder caused by the heterozygous loss of the *NF1* gene, resulting in overactivation of the RAS signaling pathway [28, 34]. Affecting approximately 1 in 3,000 births, NF1 presents with a broad spectrum of symptoms affecting nearly every organ. A hallmark of NF1 is the development of cutaneous neurofibromas (cNFs), benign nerve sheath tumors that form at nerve endings in the skin. Nearly all NF1 patients develop cNFs, which, despite their benign nature, are often considered the most distressing feature due to disfigurement, itching, and pain. As a result, cNFs pose a substantial clinical burden for individuals with NF1, significantly affecting their quality of life [18]. These tumors typically appear at puberty, exhibit transient growth, and their triggers remain unknown. Current treatment options are limited to surgical or laser ablation, which are often impractical due to the high tumor burden and risk of scarring [7, 18, 23]. However, advances in in vitro and animal models are improving our understanding of cNF pathogenesis and facilitating the discovery of novel therapies [30]. Notably, recent clinical trials with a topical MEK inhibitor have shown promising results, with partial tumor regression observed after one month of treatment [29].

cNFs arise from the Schwann cell (SC) lineage after biallelic loss of *NF1*. However, the precise timing of this somatic mutation and the specific SC subtype, myelinating (mSCs), non-myelinating (nmSCs), the latter including terminal SCs, remain unclear. Beyond *NF1*^*−/−*^ SCs, cNFs contain a complex microenvironment including fibroblasts, macrophages, mast cells, vascular and neuronal components, all embedded in a dense, collagen-rich extracellular matrix [23]. To further investigate cNF pathogenesis, we developed an *Nf1-KO* mouse model (*Prss56*^*Cre/+*^, *R26*^*Tom/+*^, *Nf1*^*fl/fl*^) that recapitulates cNF formation. This model targets the biallelic loss of *Nf1* and expresses a fluorescent tomato (*Tom*) reporter in *Prss56*-expressing boundary cap (BC) cells and their derivatives [27]. BCs are neural crest-derived cells located at nerve entry and exit zones that give rise to SCs that populate nerve roots and terminal nerve endings in the skin, key sites of neurofibroma formation in NF1 patients [11]. While *Nf1-KO* mice develop rare small cNFs from 1 year of age, we demonstrated that skin trauma promotes tumor formation. Building on these findings, we established a cNF-inducible model (*Nf1-KOi*) in which cNFs are induced by bite wounds in group-housed males. This model revealed that cNF development follows three distinct stages: initiation, progression, and stabilization, each characterized by specific cellular dynamics. In particular, SC proliferation and MAPK pathway activation occur during initiation and progression, whereas mature tumors contain quiescent SCs with silenced MAPK activity [9]. Using the *Nf1-KOi* model, we performed single-cell transcriptomic analysis at the progression and stabilization stages and revealed that a BC-derived SC subpopulation, identified as subepidermal glia, is likely the cell of origin for cNFs. Furthermore, we found that tumor SCs, in collaboration with fibroblasts and pericytes, contribute to fibrosis. Finally, we discovered that during the progression phase, tumor SCs overexpress set of genes that govern axonal growth and branching, likely responsible for the abnormally dense and disorganized innervation of cNFs in mice and patients.

## Materials and methods

### Animals

The following mouse lines were used and genotyped as described in the original publications: *Prss56*^Cre/Cre^, *R26*^tdTom/tdTom^; *Prss56*^Cre/Cre^, *R26*^tdTom/tdTom^, *Nf1*^flox/flox^; *Nf1*^flox/flox^; *Nf1*^+/−^; and B6D2F1 (https://janvier-labs.com/en/fiche_produit/b6d2f1-mouse/). We bred *Prss56*^Cre/Cre^, *R26*^tdTom/tdTom^, *Nf1*^flox/flox^ mice with the *Nf1*^flox/flox^ or *Nf1*^+/−^ mice to generate *Prss56*^Cre/+^, *R26*^tdTom/+^, *Nf1*^flox/flox^ (*Nf1*-KO) and *Prss56*^Cre/+^, *R26*^tdTom/+^, *Nf1*^flox/−^ (*Nf1*-KO^het^) mutants, respectively. *Prss56*^Cre/Cre^, *R26*^tdTom/tdTom^ mice were crossed with B6D2F1 mice to generate *Prss56*^Cre/+^, *R26*^tdTom/+^ controls. All animal manipulations were approved by a French Ethical Committee (10/01/2020, reference number APAFIS#25551-2020031115016451 v6) and were performed according to French and European Union regulations.

### Human tissue specimens

A total of 16 cNFs were surgically removed and collected from the forearm of individuals with NF1. Of these cNFs, nine were classified as sessile, five as globular, and two as flat. 4 The cNFs were defined as small (diameter < 5 mm) and 12 as medium (5 mm < diameter < 10 mm). The study was authorized by the ethical committee of the Henri-Mondor Hospital (ID RCB: 2019-A01100-57/SI:19.09.05.52652) and all participants provided their written informed consent.

### Housing conditions

Mice used in this study were housed in a temperature- and humidity- controlled vivarium with a 12-hour dark/light cycle and free access to food and water at the IMRB pathogen-free facility. To investigate the mechanisms that initiate the development of cNFs, males were separated at weaning (1 month, 1 male/cage) and then until sacrifice. To obtain growing cNFs, males were housed in groups with 3 − 5 siblings/cage and then until sacrifice. Stabilized cNFs were obtained from males that were housed in groups (3 − 5 males/cage), and then separated (1 male/cage) 1 week prior to the experiment.

### Induced skin incision procedure

P45 males kept alone since weaning were anesthetized with isoflurane and their dorsal skin was shaved and sterilized with 70% ethanol. A 5% lidocaine cream was then applied to the whole dorsal skin for 2 min. The 5 mm-long full-thickness incisional skin lesions (2 per mouse) were made with scissors in an area filled with tomato + traced cells. The mice were monitored for the following days to ensure that no infection occurred at the injury site. Animals were sacrificed on Days 6, 8, and 14 (*n* = 2 mice/stage, *N* = 2 incisions/mouse) post-injury, and their skin was harvested and processed for immunohistochemistry analysis.

### EdU labeling

P45 male mice, kept alone since weaning, were injected intraperitoneally with EdU at a dose of 20 µg/g of body weight twice a day for 4 consecutive days starting on postnatal day P45 and harvested 72 h later. Five *Nf1*-KO and four control mice were sacrificed, and their skin was dissected and fixed for 2 h at 4 °C in 4% PFA. EdU detection was performed on 14 μm-thick cryosections using the Click-IT Plus EdU Alexa Fluor 488 Imaging Kit (Thermo Fisher Scientific) according to the manufacturer’s instructions, followed by immunolabeling for tomato.

### Macroscopic analysis

For the macroscopic analysis of the presence of cNFs, mice were anesthetized, and their back skins were shaved. The dorsal skins were imaged using the ϕ-eye™ live optical imaging device from the Bioemtech company. Image processing and analysis were performed using Image J software.

### Skin processing for immunohistochemistry

Animals were shaved, depilated, and perfused transcardially (except for manually injured skins) with 50mL PBS to remove blood. For sectioning, dorsal skins of Nf1-KOi mice were fixed in 4% paraformaldehyde (PFA) overnight at 4 °C, then washed with PBS, dehydrated in 30% sucrose overnight at 4 °C, embedded in gelatin/sucrose (PBS/15% sucrose/7.5% gelatin), and frozen on dry ice. Samples were sectioned at 14 μm thickness on SuperFrost Plus slides. IHC was performed on mouse frozen sections using the following primary antibodies: rat anti-tomato (1:1000; Kerafast #EST203), rabbit anti-RFP (1:1000; Rockland #600-401-379), goat anti-CD45 (1:100; Bio-Techne #AF114), goat anti-PDGFRα (1:100; R&D system #AF1062), rabbit anti-Ki67 (1:200; Abcam #ab15580), rabbit anti-P-ERK (1:250; Cell Signaling #9101), goat anti-cKIT (1:100; R&D system #AF1356), rat anti-F4/80 (1:400; Abcam #ab6640), rat anti-MBP (1:200; Chemicon Millipore #MAB386), goat anti-POSTN (1:400; BioTechne #AF2955), rabbit anti-TNC (1:100; Abcam #ab108930), rabbit anti-PGP9.5 (1:100; Abcam #ab108986), rabbit anti-nestin (1:100; Abcam # ab221660), mouse A488-anti-Aqp1 (1:100; Santa Cruz Biotech #sc-25287). IHC was performed on frozen sections of patient cNFs using the following primary antibodies: rabbit anti-PGP9.5 (1:100; Abcam #ab108986), mouse anti-S100β (1:300; Abcam #ab218516). Fluorophore-conjugated secondary antibodies were purchased from Jackson Immuno Research. Briefly, sections were blocked with 10% normal donkey serum and 0.3% Triton X-100 in PBS for 2 h at room temperature. Sections were incubated with the primary antibodies in the same blocking solution overnight at 4 °C, then washed and incubated with the secondary antibodies in 1% normal donkey serum, 0,3% Triton X-100 in PBS solution at room temperature. Sections were counterstained with Hoechst 33,342 to detect nuclei and mounted in Fluoromount-G (Southern Biotech).

For wholemounts, dorsal skins from *Nf1*-KOi mice were fixed in 4% paraformaldehyde (PFA) for 2 h at 4 °C and washed with PBS. The samples were then cleared with Rapiclear^®^ 1.52 reagent (SunJin Lab, #RC152001) and mounted in the same reagent.

### Imaging and immunostaining qualitative and semiquantitative analyses

Images were captured with a Zeiss LSM 900 confocal microscope equipped with a 20X objective. They were acquired in the.czi format before being processed and analyzed using Image J software.

For all semi-quantitative analyses, raw full-stack images were stacked, processed to remove autofluorescent background, saved as.tif images, and automatically processed for the proportion of tomato + SCs and MBP + myelinating SCs using specific Image J macros. Results were processed in GraphPad Prism 10 software and presented as mean +/- SEM.

### Skin processing for single cell RNA sequencing

#### 3-hour dissociation protocol

Animals were anesthetized, shaved, depilated, and perfused transcardially with 50mL PBS to remove any blood. Their skin was carefully dissected, and adipose tissue was removed with scalpels, which was supplemented by three successive washes in 30mL RPMI. Each piece of skin was then carefully placed in a petri dish, epidermis side down, and treated with 15mL of 0.15% trypsin-EDTA for another 30 min at 37°. After this incubation, the skins were removed from the trypsin-EDTA solution and placed in a PBS/5%FBS solution at 4 °C, where the detached epidermis was gently scraped off with scalpels. The skins were then washed three times in 30mL of RPMI before being exposed to the trypsin-EDTA medium for another 30 min at 37 °C. After this second incubation, the enzymatically dissociated epidermis was scraped off using the same procedure. After a final rinse with RPMI, the skins were carefully dissected under a Leica epi-fluorescence microscope to isolate cNFs and HLS regions, which were then finely minced on a -20 °C metal block using scissors and scalpels. The minced tissue (0.5 g per tube) was then immersed in 10mL of prewarmed RPMI medium containing Liberase DH (0.25 mg/mL) and placed in a 37 °C water bath for 3 h. To achieve complete dissociation, the skin tissue was mechanically dissociated every 30 min with a 1mL pipette. After 40 min of dissociation, DNAseI was added to the medium. After 3 h of incubation, the dissociation tubes were immediately placed on ice. Any remaining skin pieces were then mechanically dissociated using an up-and-down motion, first with a 1mL pipette and then with a glass Pasteur pipette precoated with FBS. To stop the action of the enzymes, 30mLof RPMI containing 10% FBS was added to each tube. The tubes were then centrifuged at 300 g for 10 min. After discarding the supernatant, the pellet was gently resuspended in 5mL RPMI and filtered through a 100 μm filter, followed by a 70 μm filter, and finally a 30 μm filter to remove any remaining tissue fragments or cellular debris. The cells were then centrifuged again at 300 g for 10 min and resuspended in a solution of PBS/1% BSA. To assess cell viability, DAPI viability dye was added at a concentration of 5 µg/mL. The prepared cells were then sorted by fluorescence-activated cell sorting (FACS) at the IMRB FACS facility. The sorting process was based on cell DAPI labeling and tomato fluorescence to ensure selection of viable and relevant cell populations.

#### 18-hour dissociation protocol

Animals were anesthetized, shaved, depilated, and perfused transcardially with 50mL PBS to remove any blood. Their skin was carefully dissected, and adipose tissue was removed with scalpels, which was supplemented by three successive washes in 30 mL RPMI. Each piece of skin was then carefully placed in a petri dish, epidermis side down, and treated with 15mL of 0.15% trypsin-EDTA for 30 min at 37 °C. After this incubation, the skins were removed from the trypsin-EDTA solution and placed in a PBS/5%FBS solution at 4 °C, where the detached epidermis was gently scraped off with scalpels. The skins were then washed three times in 30mL RPMI before being exposed to the trypsin-EDTA medium for another 30 min at 37 °C. After this second incubation, the enzymatically-dissociated epidermis was scrapped of using the same procedure. After a final rinse with RPMI, the skins were carefully dissected under a Leica epi-fluorescence microscope to isolate cNFs and HLS regions. Small pieces (2 × 2 mm) of each sample type were cut and then immersed (six pieces per well) in 2 ml-DMEM medium containing 10% FBS, 10% penicillin/streptomycin, and Liberase DH (0.25 mg/mL). The filled 6-well plate was then placed into an incubator (37 °C, 5% CO2) for 18 h overnight without shaking. The next day, to stop the action of the enzymes, 8mL DMEM medium containing 10% FBS was added to each well, which was followed by gentle mechanical dissociation with a 1mL pipette. The tubes were then centrifuged at 300 g for 10 min. After discarding the supernatant, the pellet was gently resuspended in 5mL of DMEM and filtered through a 100 μm filter, which was followed by a 70 μm filter, and finally a 30 μm filter to remove any remaining tissue fragments or cellular debris. The cells were then centrifuged again at 300 g for 10 min and resuspended in a solution of PBS/1% BSA. To assess cell viability, DAPI viability dye was added at a concentration of 5 µg/mL. The prepared cells were then sorted using fluorescence-activated cell sorting (FACS) at the IMRB FACS facility. The sorting process was based on DAPI labeling of the cells to ensure the selection of viable cell populations.

### Single-cell RNA sequencing

Approximately 25,000 cells were loaded into one channel of the Chromium system using the V3.1 single-cell reagent kit (10X Genomics) to generate single-cell GEMs. After capture and lysis, cDNAs were synthesized, and then amplified by PCR for 12 cycles according to the manufacturer’s protocol (10X Genomics). The amplified cDNAs were used to generate Illumina sequencing libraries, each of which was sequenced on a Nextseq500 Illumina flow cell.

### Bioinformatic analyses

BCL files were processed using 10x Genomics Cell Ranger 7.0.1. Reads were mapped to a custom mouse transcriptome based on the GRCm39 release 108 transcriptome, to which we added sequences such as *tdTomato*. For both the custom transcriptome construction and the BCL to count matrix pipeline, we designed two pipelines using Nextflow [10] and Singularity containerization [17]. Count matrices were processed from raw matrices to the figures using RStudio and Rmarkdown in order to generate fully traceable notebook for each analysis. To ensure version stability, a singularity container containing R version 3.6.3 (10.5281/zenodo.13145083) and all specific versions for all the packages of interest was developed and used to compile notebooks.

Each data set, which corresponded to one biological replicate from one experimental condition, was analyzed individually from its raw count matrix using the Seurat V3 package [29]. Cells with less than 2^6^ UMIs or less than 600 genes were filtered out. Doublet cells were removed using the scDblFinder tool and scds in hybrid mode. Then, cells with more than 20% of UMI related to mitochondrial genes or more than 40% related to ribosomal genes were filtered out. The UMI count matrix for the remaining cells was normalized using the LogNormalize method implemented in the Seurat V3 package. From the normalized count matrix, we used AddModuleScore to annotate cells for cell types using specific marker sets. Finally, cells were clustered using Seurat’s FindClusters function with a resolution of 1, on 20 principal components of a 100-dimensional PCA obtained using Seurat’s RunPCA function from 3,000 highly variable features.

The same pipeline was used to combine either of all 12 whole skin data sets or all 11 cutaneous tomato + data sets. The data sets of interest were merged using base’s merge function. Genes expressed in fewer than five cells were removed. PCA was performed on the individual data sets. Sampling effect was removed on the PCA using the RunHarmony function from the harmony package [15, 16]. To generate UMAP, the function RunUMAP from the Seurat Package was used. Cells were clustered on the harmonized PCA. To smooth out possible mis-annotation, cell type annotations were grouped by cluster.

To same procedure was used to build the four-cell type-related data sets. Cells were extracted from each individual data set based on single cell or cluster smoothen annotation. After the first processing as for the atlas, possible contaminating cells were automatically removed by clustering and marker genes. A second processing was applied to selected cells. For each cell type-related data set, the sample effect was removed from the normalized count matrix using the harmony package, with UMAPs obtained. Cells were clustered on the corrected space and re-annotated. Annotation was smoothed at the cluster level. For differential expression, the Wilcoxon test from Seurat’s FindMarkers function was used. Differentially expressed genes were filtered based on adjusted p-value less than 0.05. To assign a collagen gene score to each cell, the AddModuleScore function from the Seurat package and a gene set containing collagen-encoding genes were applied: “*Col1a1*”, ”*Col1a2*”, ”*Col2a1*”, ”*Col3a1*”, ”*Col4a1*”, ”*Col4a2*”, ”*Col4a3*”, ”*Col4a4*”, ”*Col4a5*”, ”*Col4a6*”, ”*Col5a1*”, ”*Col5a2*”, ”*Col5a3*”, ”*Col5a4*”, ”*Col6a1*”, ”*Col6a2*”, ”*Col6a3*”, “*Col6a4*”, ”*Col6a5*”, ”*Col6a6*”, ”*Col7a1*”, ”*Col8a1*”, “*Col8a2*”, ”*Col9a1*”, “*Col9a2*”, ”*Col9a3*”, ”*Col10a1*”, ”*Col11a1*”, “*Col11a2*”, ”*Col11a3*”, ”*Col12a1*”, ”*Col13a1*”, ”*Col14a1*”, ”*Col15a1*”, ”*Col16a1*”, ”*Col17a1*”, ”*Col18a1*”, ”*Col19a1*”, ”*Col20a1*”, ”*Col21a1*”, ”*Col22a1*”, ”*Col23a1*”, ”*Col24a1*”, ”*Col25a1*”, ”*Col26a1*”, ”*Col27a1*”, ”*Col28a1*”.

## Results

### Loss of *Nf1* in BC-derived Schwann cells lineage transiently promote their proliferation

**Fig. 1:**
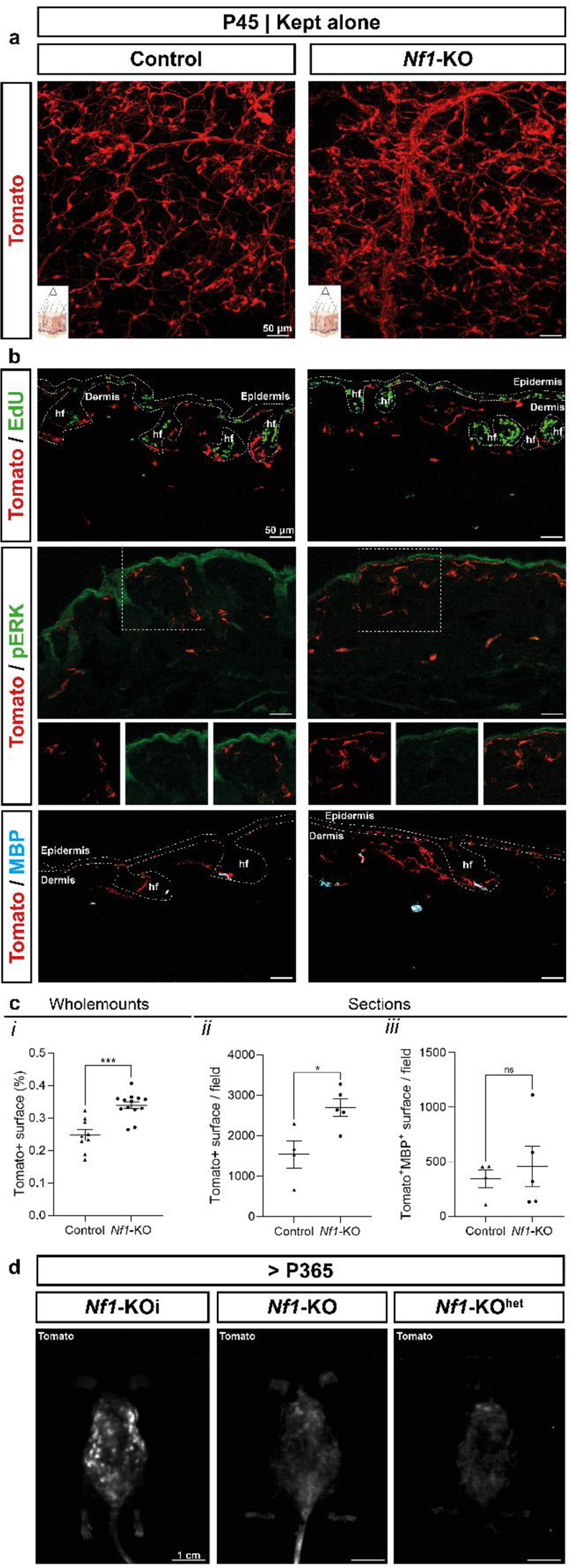
Effects of *Nf1* loss in BC-derived cutaneous SCs. (**a**) Wholemounts of young (P45) control and *Nf1*-KO mouse skins showing distribution of Tom^+^ BC-derived Schwann cells (Tomato^+^). (**b**) Immunostaining showing respectively Tom^+^ BC-derived Schwann cells (Tomato^+^), dividing cells (EdU^+^), pERK + cells (pERK^+^) and myelinating SCs (MBP^+^) in P45 control and *Nf1*-KO skins. (**c**) Quantification of the Tom + surface per field in wholemounts (***i***) sections (***ii***) and of Tomato^+^MBP^+^ surface per field in sections (***iii***). (**d**) Macroscopic fluorescence images of the dorsal skin of grouped *Nf1*-KOi and isolated *Nf1*-KO and *Nf1*-KO^het^ mice. Scale bars: 50 μm (**a**, **b**), 1 cm (**d**). hf: hair follicles. P45: Postnatal Day 45. P365: Postnatal Day 365. SCs: Schwann cells. BC: Border capsules. cNFs: cutaneous neurofibromas. TOG: grouped. dpi: days post injury

We investigated how loss of *Nf1* in BC-derived Schwann cells (SCs) influences their proliferative activity in young (P45) *Nf1-KO* mice before the onset of cNFs. In mutant skin, the number of SCs was 37% higher than in controls; however, they remained quiescent and showed no MAPK activation, as indicated by EdU incorporation and pERK staining (Fig. 1a-c). This suggests that mutant SCs likely underwent a transient proliferative phase before this time point, consistent with our previous findings of increased SC proliferation around birth [25].

To determine whether the increased SC population consisted primarily of mSCs or nmSCs, we performed myelin (MBP) staining on mutant and control skin at P45. Mutant nerves showed a higher proportion of nmSCs (MBP⁻,Tom⁺), whereas the proportion of mSCs (MBP⁺,Tom⁺) remained unchanged compared to controls (Fig. 1a-c). This suggests that loss of *Nf1* transiently stimulates proliferation specifically in the nmSC lineage, without affecting mSCs. Despite this excess of mutant nmSCs, none of the *Nf1-KO* mice developed cNFs prior to 1 year of age in the absence of trauma. This indicates that skin trauma, in combination with loss of *Nf1*, is essential for cNF development in this model (Fig. 1d).

### A single-cell resolution transcriptomic atlas of growing and mature mouse cNFs

To elucidate the cellular and molecular landscape driving the development of cNF in mice, we performed single-cell RNA sequencing (scRNAseq) on cNFs, adjacent healthy-looking skin (HLS), and control healthy skin (HS) at both the growth (P90) and stabilization (P365) stages. To optimize cell dissociation while preserving transcriptomic integrity, we compared a 3-hour versus an 18-hour (overnight) dissociation protocol. The overnight protocol yielded a higher proportion of non-keratinocyte cells (41–43% at 3 h vs. 68–70% at 18 h), including SCs (2–3% vs. 8–10%, respectively; Supplementary Fig. 1a-c). However, transcriptomic comparisons revealed protocol-dependent differences in SCs and nerve fibroblasts (Supplementary Fig. 1d-e). Specifically, more genes were upregulated in cNFs relative to HLS in the 3-hour condition than in the 18-hour condition in both SCs (530 vs. 270 genes) and fibroblasts (285 vs. 70 genes). Notably, fibroblasts exhibited reduced collagen gene expression in cNFs compared to HLS when using the 18-hour protocol (collagen gene expression score: 0.21 in the 3-hour protocol vs. -0.07 in the 18-hour protocol). These results suggest that while prolonged dissociation improves cellular representation, it compromises transcriptomic integrity, leading to homogenization with HLS profiles. On this basis, we selected the 3-hour dissociation protocol for this study.

**Fig. 2:**
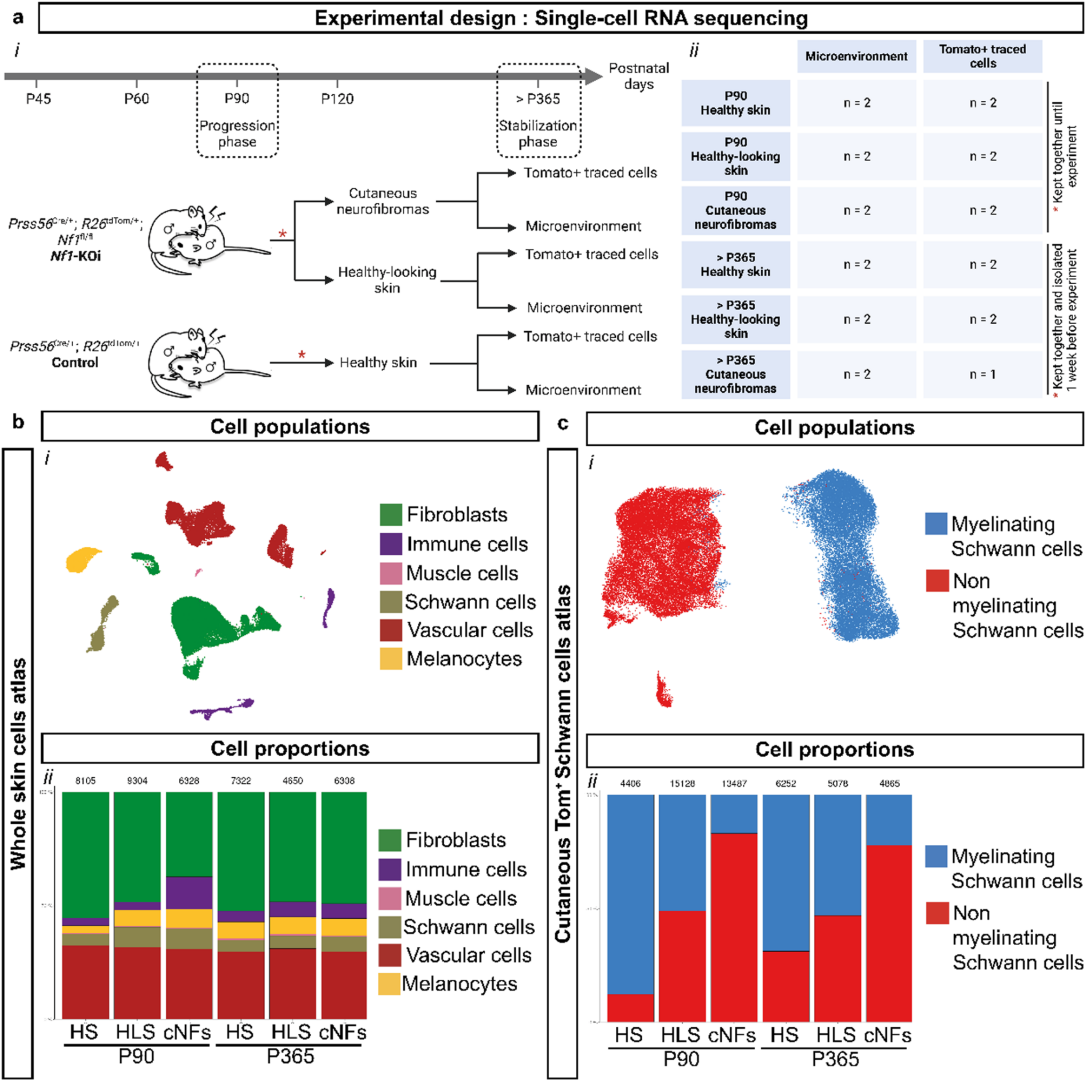
Single-cell transcriptomic atlas of murine cutaneous neurofibromas. (**a*i***) Schematic overview detailing the experimental workflow used to generate the scRNAseq data set. (**a*ii***) Comprehensive summary recapitulating the number of scRNAseq experiments performed and the housing conditions of the mice. (**b**) UMAP of whole skin cell types: (***i***) visualization of cell populations after the removal of keratinocytes. (***ii***) proportional distribution of cell types across various experimental conditions from the atlas. (**c**) UMAP of cutaneous Tom + SCs: (***i***) UMAP highlighting myelinating and non-myelinating SC populations after the removal of melanocytes. (ii) Proportional distribution of Tom + SC subtypes in different conditions from the atlas. P90: Postnatal Day 90. P365: Postnatal Day 365. cNFs: cutaneous neurofibromas. HLS: healthy appearing skin. HS: healthy skin. SCs: Schwann cells

Our scRNAseq analysis included both control and *Nf1-KOi* mice. Control mice provided data from HS, while mutant mice provided data from cNFs and adjacent HLS. Because Tom⁺ SCs comprised a small fraction of total skin cells (≤ 4% in cNFs, ≤ 1.5% in HS, as determined by FACS; Supplementary Fig. 1f), we performed scRNAseq on both whole skin cells and FACS-isolated Tom⁺ cells for each sample type. To minimize inflammation-related variability, mutant male mice were isolated for 1 week prior to sacrifice in stabilization phase experiments to ensure that analyses focused on cNF-specific changes rather than injury-induced effects (Fig. 2a, M&M). Two different scRNAseq atlases were generated. The first, a whole skin dataset, included all major skin cell types after enzymatic and computational exclusion of keratinocytes. The second, a Tom⁺ SC dataset, focused on Tom⁺ SCs after computational removal of infrequently detected melanocytes. Cell types were annotated using specific markers, and the presence of tomato reporter expression in the Tom⁺ SC dataset was confirmed (Supplementary Fig. 2a-b). The whole skin dataset identified fibroblasts, muscle cells, SCs, melanocytes, vascular cells, and immune cells. As expected, the Tom⁺ SC data set revealed distinct mSC and nmSC populations.

Cellular composition varied between conditions in both data sets (Fig. 2b-c). In the whole skin data set, the proportion of immune cells increased in growing cNFs, consistent with previous IHC observations of acute inflammation at this stage. In the Tom⁺ SC data set, the proportion of Tom⁺ nmSCs progressively increased from HS and HLS to cNFs in both growth and stabilization phases (Fig. 2c).

Taken together, we have generated the first comprehensive transcriptomic atlases of dermal skin cells and Tom⁺ SCs derived from mouse cNFs, along with their corresponding control tissues.

### Aqp1^high^ Nes^low^ nmSCs are likely population of origin of cNFs

**Fig. 3:**
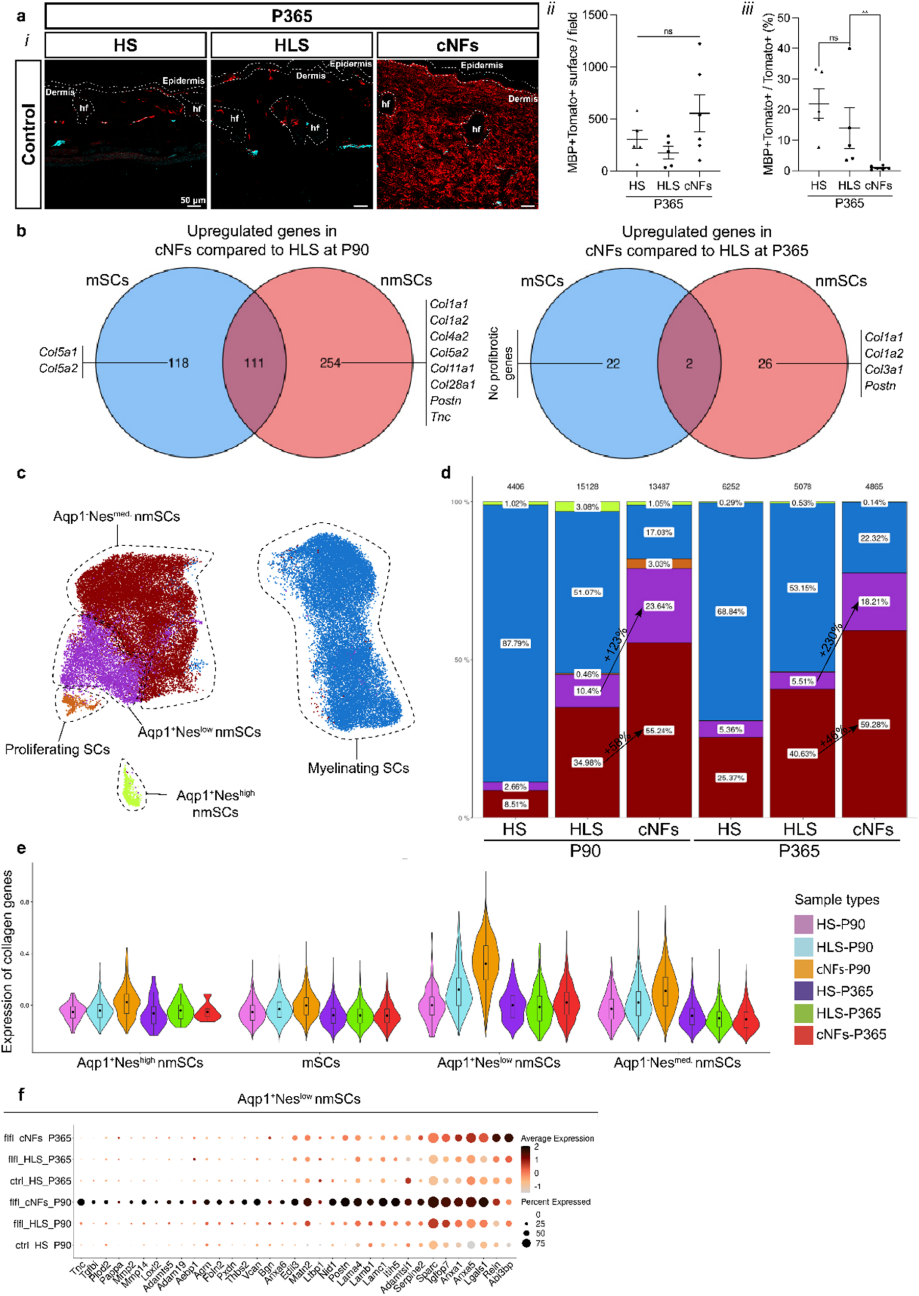
Profiling of Tom + SCs from mouse cNFs. (**a*i***) Labeling of myelinating (MBP^+^) and Tom^+^ SCs (Tomato^+^) from the HS, HLS and cNFs at P365. (**a*ii***) Quantification of the MBP^+^Tom^+^ labeling surface per field and (**a*iii***) MBP^+^Tom^+^/Tom^+^ labeling area ratio in the HS, HLS, and cNFs at P365. (**b**) Venn diagram showing the upregulated genes in cNFs compared to the HLS at P90 and P365 in mSCs vs. nmSCs. (**c**) UMAP plot of the subpopulations of Tom^+^ SCs. (**d**) Cell proportions of the different Tom + SCs subpopulations across the different conditions. (**e**) Violin plot showing collagen genes expression in Tom + SCs subpopulations in the different conditions. (**f**) Dot plot showing the matrisome-related signature of the Aqp1^+^Nes^low^ SC subpopulation in the different conditions. SCs: Schwann cells. nmSCs: non-myelinating Schwann cells. mSCs: myelinating Schwann cells. cNFs: cutaneous neurofibromas. HLS: healthy appearing skin. HS: healthy skin. P90: Postnatal Day 90. P365: Postnatal Day 365. Scale bars: 50 μm

Given the established role of the SC lineage in the origin of cNFs, our initial focus was on profiling this population. The data set included mSCs and nmSCs (Fig. 2b). As noted above, the proportion of nmSCs increased in growing and mature cNFs. IHC analysis confirmed this trend, showing a higher proportion of Tom + nmSCs in cNFs compared to their HLS or HS, while the proportion of Tom + mSCs remained consistent with that of their corresponding HLS or HS (Fig. 3a). These results suggest that nmSCs are the primary cellular component of cNFs, while mSCs are unlikely to play a role in their development. To further investigate the contribution of each SC population towards cNF pathogenesis, we identified differentially expressed genes in mSCs and nmSCs in the growing and mature cNFs relative to their adjacent HLS. A subset of collagen-encoding genes was specifically upregulated in nmSCs, reaffirming their pivotal role in cNF development (Fig. 3b). Given these findings, we focused on Tom + nmSCs derived from cNFs in the growth and stabilization phases. Transcriptomic analysis revealed multiple nmSC subpopulations, each characterized by unique marker expression patterns (Fig. 3c, Supplementary Fig. 3a-b). Among these, aquaporin1 (Aqp1) and nestin (Nes) emerged as key markers for distinguishing nmSC subtypes. Based on their expression levels, four distinct subpopulations were identified: (i) Aqp1^high^Nes^high^, (ii) Aqp1^high^Nes^low^, (iii) Aqp1^low^Nes^med^ and (iv) Aqp1^low^Nes^low^ (proliferating nmSCs marked by *Mki67*, *Birc5*, and *Top2a*, indicating growth of cNFs [8]). To align these subpopulations with known cutaneous nmSC subtypes, including lanceolate glia, subepidermal glia, arrector-pili glia and classical nmSCs, we performed AQP1 and NESTIN immunostaining in young control skin (P45). This analysis identified: (i) TOM + AQP1^high^NESTIN^low^ multipolar cells predominantly at the epidermis-dermis junction, likely corresponding to subepidermal glia, (ii) TOM + AQP1^high^NESTIN^high^ cells near hair follicles, representing lanceolate and arrector-pili glia and (iii) TOM + AQP1^low^NESTIN^med^ cells aligned with deeper dermal nerves, corresponding to classical nmSCs (Supplementary Fig. 3c). We then assessed the relative abundance of these nmSC subtypes in cNFs compared to adjacent HLS or control HS at different disease stages. A significant enrichment of Aqp1^high^Nes^low^ (+ 123% in growing cNFs, + 230% in mature cNFs) and Aqp1^low^Nes^med^ (+ 58% in growing cNFs, + 46% in mature cNFs) populations was observed, supporting their involvement in cNF development (Fig. 3d).

Since fibrosis accounts for almost half of the cNF volume and is mainly composed of collagen, we examined the profibrotic activity of these SC subpopulations. The Aqp1^high^Nes^low^ SCs exhibited increased expression of collagen-related genes compared to other SC populations (Fig. 3e). During the stabilization phase, these cells expressed higher levels of collagen types I, III, and V in cNFs relative to HLS (Supplementary Fig. 3d). During the progression phase, a broader spectrum of collagen genes was upregulated including: Aqp1^high^Nes^low^: Collagen types I, III, IV, V, VI, VIII, XI, XV, XVIII, XXVIII and Aqp1^low^Nes^med^: Collagen types I, III, V, VIII, XI, XII, XVIII, XXVIII (Supplementary Fig. 3d). Given their enrichment in cNFs and increased profibrotic activity, we propose that Aqp1^high^Nes^low^ SCs could be the cells of origin. To further investigate this hypothesis, we analyzed the transcriptomic changes in growing and mature cNFs compared to HLS or HS. This revealed an upregulation of several matrisome-associated genes, including *Postn*,* Tnc*,* Tgfbi*, and *Abi3bp* (Fig. 3f).

### cNFs show dense and disorganized innervation pattern

**Fig. 4:**
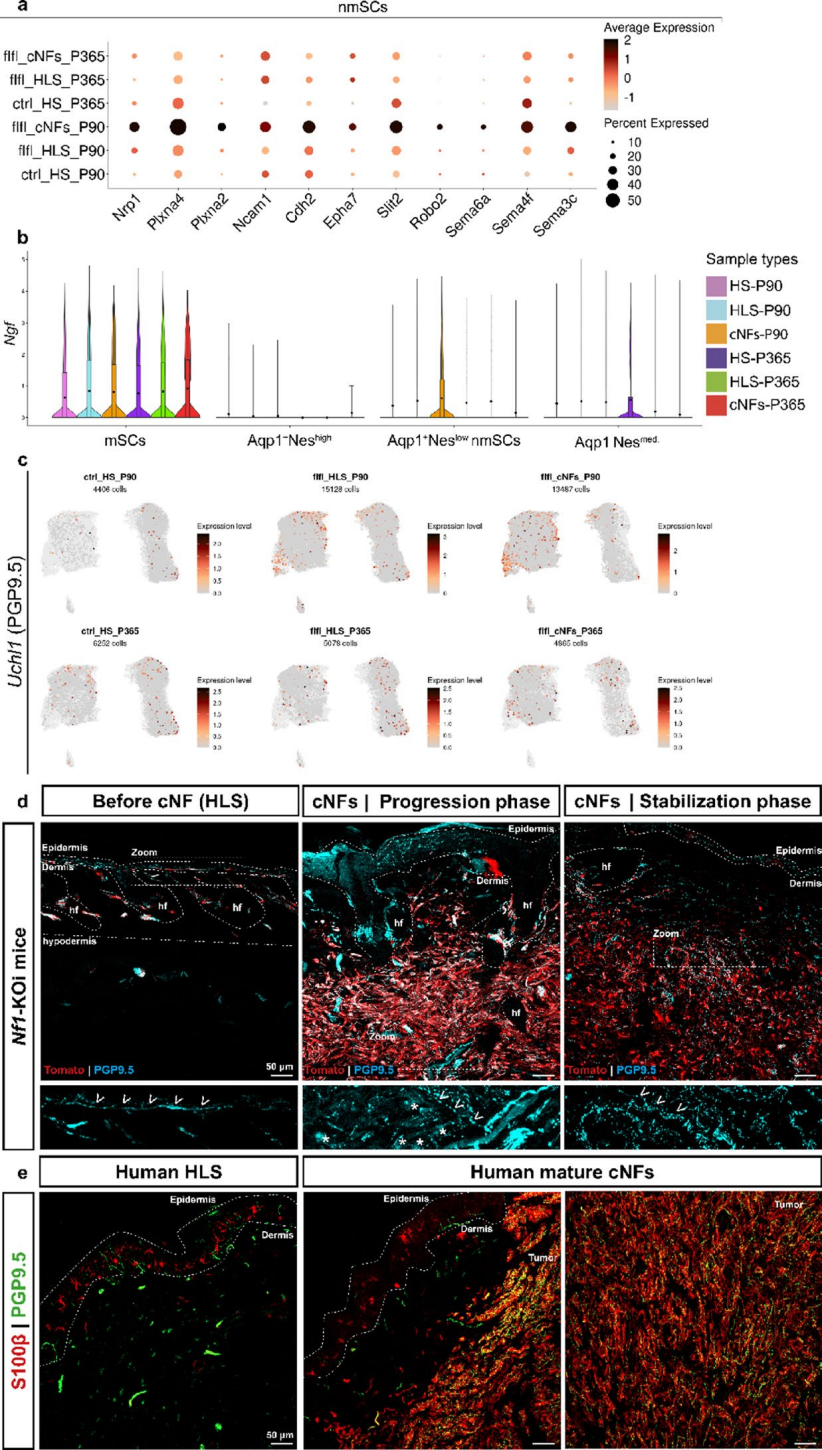
Mutant SCs express genes that regulate axon growth and branching. (**a**) Dot plot showing the overexpression by mutant SCs of genes that regulate axongrowth and branching of growing (P90) cNFs. (**b**) Violin plot showing the expression of *Ngf* gene in the Tom^+^ SCs data set in the different conditions. (**c**) Feature plot showing the expression of *Uchl1* encoding the neuronal marker PGP9.5 in the Tom^+^ SCs data set in the different conditions. (**d**) Immunofluorescence showing accumulation of Tom^+^*Nf1* mutant SCs and dense (PGP9.5^+^) innervation of developing (P90) and mature (P365) cNFs from *Nf1*-KOi mice. (**e**) Immunofluorescence showing accumulation of SCs (S100β^+^) and abnormal density of axons (PGP9.5^+^) in cNFs from patients compared to HLS. SCs: Schwann cells. nmSCs: non-myelinating Schwann cells. mSCs: myelinating Schwann cells. cNFs: cutaneous neurofibromas. HLS: healthy appearing skin. HS: healthy skin. P90: Postnatal Day 90. P365: Postnatal Day 365. Scale bars: 50 μm

We observed that *Nf1*-mutant nmSCs in growing cNFs specifically express genes involved in axon guidance, growth, and branching compared to HS, HLS, and mature cNFs (Fig. 4a). This gene expression profile includes both axon repulsion and axon attraction functions. Within this population, Aqp1^high^Nes^low^ SCs overexpress genes encoding the neurotrophic factor NGF and the pan-neuronal marker UCHL1 (PGP9.5) (Fig. 4c). This “axon guidance and growth” signature prompted us to investigate cNF innervation in mutant mice and NF1 individuals.

We first performed IHC on growing and mature cNFs and HLS in mice using PGP9.5. During the growing phase, cNFs exhibited an unusually dense and disorganized network of PGP9.5 + axons compared to HLS, along with Tom + SCs expressing this marker, confirming our scRNAseq findings. This aberrant axonal phenotype persisted into the stabilization phase, indicating a persistent, pathological innervation of cNFs (Fig. 4d). Next, we analyzed cNF innervation in 16 NF1 patients using PGP9.5 in combination with the SC marker S100β. As a control, we examined HLS from the contralateral skin side. All 16 cNFs showed a dense and disorganized axonal network (Fig. 4e). Because PGP9.5 is a pan-neuronal marker, we could not determine the specific neuronal subtypes that innervate cNFs. However, the close contact between tumor SCs and axons suggests that almost all mutant SCs remain associated with the extra-numerical axons in cNFs. This is in contrast to plexiform NFs, where tumor SCs are reported to detach from axons [33]. Collectively, our findings demonstrate that cNF development is associated with abnormally dense and disorganized innervation. Tumor SCs express genes involved in axon guidance and growth, which could contribute to this phenotype.

### Dermal and nerve-associated fibroblasts contribute to fibrosis in cNFs

**Fig. 5:**
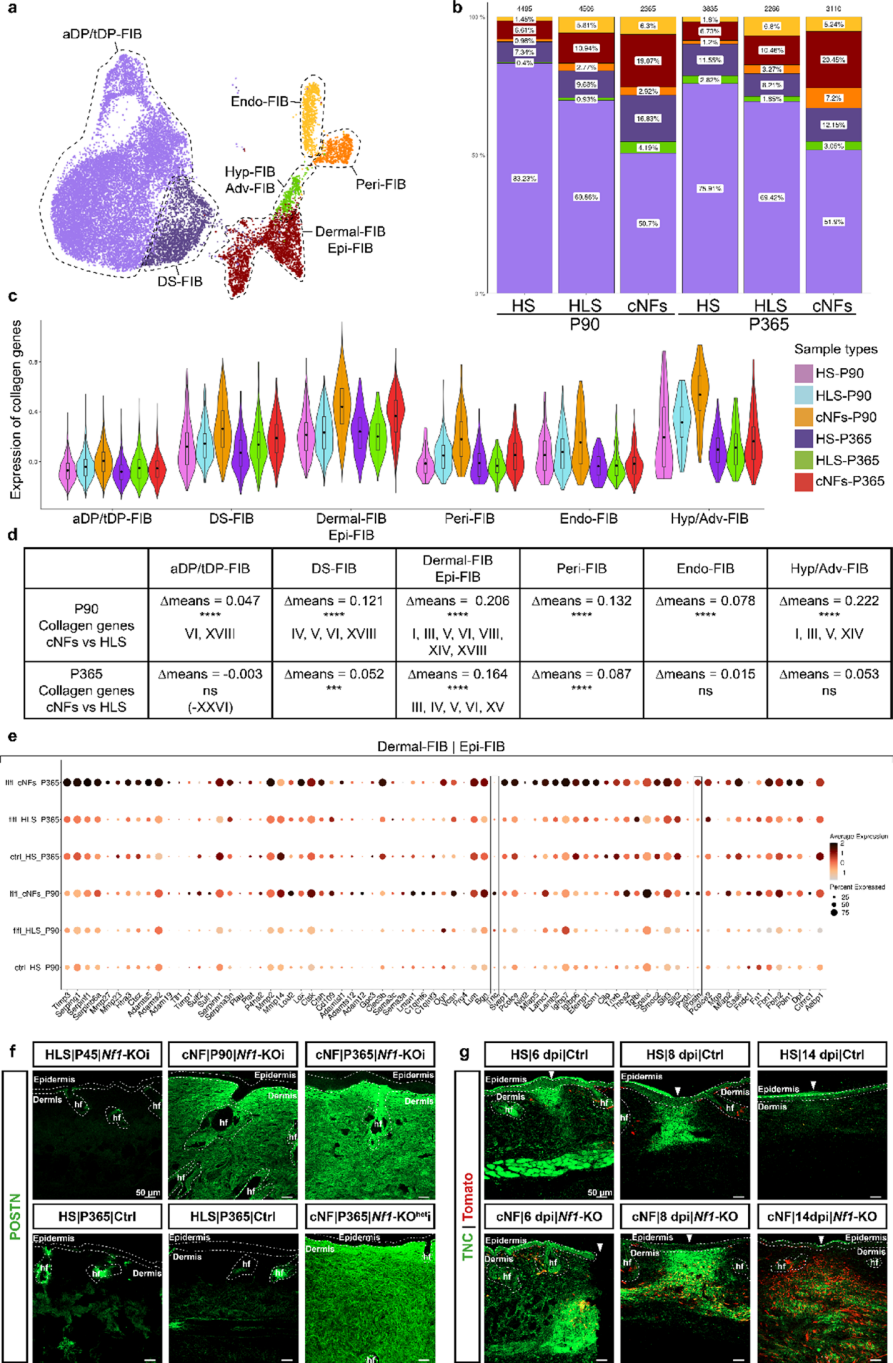
Characterization of fibroblast subpopulations and ECM from cNFs. (**a**) UMAP plot of different fibroblasts populations in the different conditions. (**b**) Distribution of the different fibroblast subpopulations in the different conditions. (**c**) Violin plot showing global collagen gene expression in the different conditions and fibroblast subpopulations. (**d**) Table recapitulating the differential expression of specific collagen genes between the cNFs and HLS in the different conditions and fibroblast subpopulations. (**e**) Dot plot showing the matrisome-related signature of the dermal| epineurial fibroblasts subpopulation between cNFs, corresponding HLS and HS from growing and mature cNFs. (**f**) Immunofluorescence showing the accumulation of POSTN (POSTN^+^) in growing and mature cNFs from *Nf1*-KOi and *Nf1*-KOi^het^ mice compared to the corresponding HLS or HS. (**g**) Immunofluorescence showing the sustained presence of TNC (TNC^+^) in injury-induced cNFs of *Nf1*-KOi mice compared to the injured corresponding HS. dpi: days post injury. cNFs: cutaneous neurofibromas. HLS: healthy-looking skin. HS: healthy skin. P90: Postnatal Day 90. P365: Postnatal Day 365. aDP/tDP-FIB: anagen and telogen dermal papilla fibroblasts. DS-FIB: dermal sheath fibroblasts. Hyp/Adv-FIB: hypodermal/adventitial fibroblasts. Endo-FIB: endoneurial fibroblasts. Peri-FIB: perineurial fibroblasts. Dermal-FIB| Epi-FIB: dermal and epineurial fibroblasts. Scale bars: 50 μm

Fibroblasts (Fb) are the second major cellular component of cNFs and a major source of fibrosis. To better understand their role, we analyzed Fb within the whole skin data set. First, Fb were extracted and annotated using specific markers (Supplementary Fig. 4a) [12], allowing for the identification of multiple Fb populations, including dermal papilla (anagen DP| telogen DP-FIB), dermal sheath (DS-FIB), hypodermal/adventitial fibroblasts (Hyp-FIB| Adv-FIB), as well as endoneurial (Endo-FIB) and perineurial Fbs (Peri-FIB). Because it was difficult to distinguish between dermal and epineurial Fbs, these groups were combined into a single population (Dermal-FIB| Epi-FIB) (Fig. 5a).

Analysis of Fb proportions in different conditions demonstrated significant changes (Fig. 5b). In mutant HLS compared to HS, the proportion of Endo-FIB increased from 1.5% to ~ 6%, Peri-FIB from 1% to ~ 3%, and Dermal| Epi-FIB from 6.5 to 11%. In growing cNFs (P90), the proportion of Dermal| Epi-FIB further increased to 19%, DS-FIB from 10 to 17%, and Hyp-FIB| Adv-FIB from 1 to 4%. In mature cNFs (P365), these differences persisted, with Dermal| Epi-FIB increasing to 20% and Peri-FIB to 7%. Taken together, dermal and nerve-associated Fb populations were more abundant in growing and mature cNFs compared to HS. However, no evidence of increased proliferation was observed in these populations based on the expression of *Mki67*, *Birc5*, (data not shown), and *Top2a* (Supplementary Fig. 4c). Analysis of collagen gene expression revealed an upregulation in all Fb populations in growing cNFs (Fig. 5c-d). This “fibrogenic” signature became more selective in mature cNFs (P365), where collagen gene activation was restricted to Dermal| Epi-FIB, DS-FIB, and Peri-FIB. The sustained increase in collagen expression in Dermal| Epi-FIB throughout cNFs development suggests that this population plays a key role in cNFs fibrosis. Further transcriptional analysis of Dermal| Epi-FIB revealed the upregulation of matrisome-related genes, along with *TIMPs* and *MMPs*, which regulate extracellular matrix remodeling in growing and mature cNFs (Fig. 5e). Unexpectedly, two matrisome-related genes, *Postn* and *Tnc*, were co-activated in both Dermal| Epi-FIB and Aqp1^high^Nes^low^ nmSC populations. While Postn was elevated in Dermal| Epi-FIB cells in both growing and mature cNFs, *Tnc* upregulation was restricted to growing tumors. Both POSTN and TNC are glycoproteins involved in fibrosis through activating fibroblasts and stimulating collagen production [3, 21]. In addition, POSTN promotes inflammation [2] and itching [20], while TNC has been associated with abnormally dense skin innervation [5] that is characteristic of cNFs. To assess the expression of POSTN and TNC in mouse cNFs, we performed IHC. Increased levels of POSTN were detected in both growing and mature cNFs compared to HLS or HS. POSTN was also observed in cNFs from *Nf1*-KOi^het^ mice, indicating that *Nf1* heterozygosity in the cNFs microenvironment does not affect their development (Fig. 5f). To investigate TNC expression, we used a skin trauma model in *Nf1-KO* mice, where a small incision promotes local cNFs growth within two weeks [25]. TNC levels were assessed at 6-, 8-, and 14 days post-injury (dpi). At 6 and 8 dpi, TNC expression was increased in both Nf1-KO and control skin. However, by 14 dpi, TNC expression remained elevated in the mutant but decreased sharply in controls (Fig. 5g). These results reveal a persistently high expression of both POSTN and TNC during cNFs development, underscoring their critical roles in this process.

### Pericytes contribute to ECM remodeling of cNFs

**Fig. 6:**
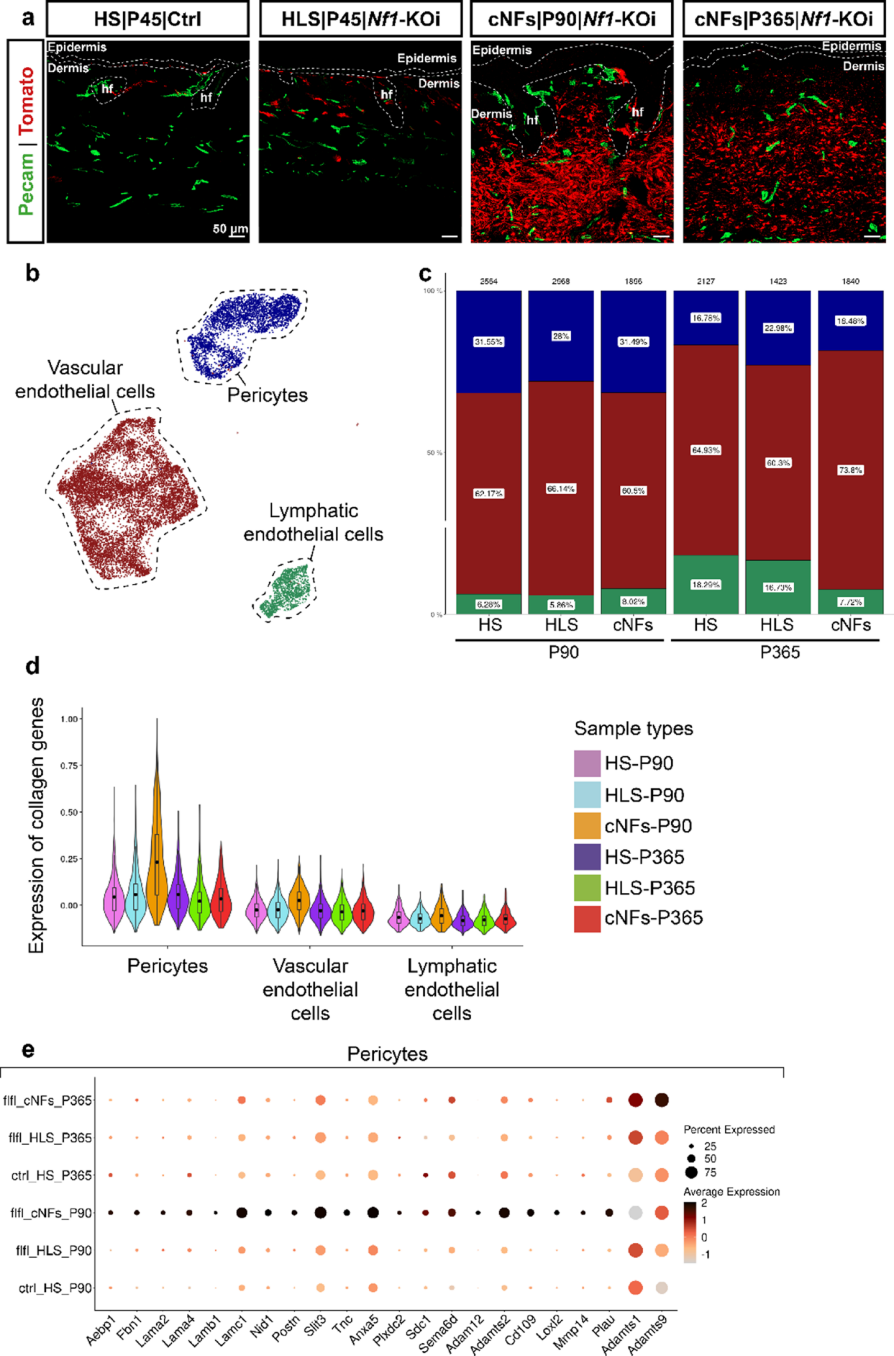
Pericytes contribute to ECM production of cNFs. (**a**) Immunofluorescence showing vascular cells (PECAM^+^) and Tom^+^ SCs in P45 HS and HLS, growing (P90) and mature (P365) cNFs. (**b**) UMAP plot of different types of vascular cells. (**c**) Distribution of the different vascular cell populations across conditions. (**d**) Violin plot showing global collagen gene expression in the different conditions and three major vascular cell populations. (**e**) Dot plot showing the signature of pericytes in growing and mature cNFs compared to the corresponding HLS. SCs: Schwann cells. cNFs: cutaneous neurofibromas. HLS: healthy appearing skin. HS: healthy skin. P45: Postnatal Day 45. P90: Postnatal Day 90. P365: Postnatal Day 365

To investigate the contribution of vascular cells towards cNF development, we performed vasculature staining using the endothelial marker Pecam on HS, HLS, and cNFs (Fig. 6a). Vascular density in cNFs remained unchanged throughout the growth and maturation phases compared to HLS, except for a modest increase in growing cNFs compared to HS (Supplementary Fig. 5a). Using the whole skin cell atlas, we analyzed the transcriptomic profiles and proportions of vascular cells in cNFs by extracting and annotating each cell type using specific markers [13] (Supplementary Fig. 5b). This analysis identified pericytes, vascular endothelial cells and lymphatic endothelial cells (Fig. 6b). Consistent with the IHC findings, the proportions of these populations remained stable across conditions (Fig. 6c).

In growing cNFs, pericytes exhibited upregulation of collagen- and matrisome-related genes compared to HLS and HS (Fig. 6d, Supplementary Fig. 5c), suggesting their involvement in ECM remodeling (Fig. 6e). In particular, ECM proteases, including *Mmp14*, *Adam12*, and *Adamts1/2/9*, were upregulated along with basal membrane proteins, including laminins (*Lama2*, *Lama4*, *Lamb1*, *Lamc*1), nidogens (*Nid1*), and proteoglycans (*Tnc*,* Postn*,* Fbn1*) [11]. Interestingly, *Tnc* and *Postn* upregulation in pericytes mirrored expression patterns observed in Dermal| Epi-FIB fibroblasts and Aqp1⁺Nes⁻ nmSC populations. In addition, increased expression of *Loxl2*, known to stabilize collagen crosslinking within the ECM [30, 32], highlights the role of pericytes in ECM dynamics during cNF progression (Fig. 6e).

### Immune landscape of cNFs

**Fig. 7:**
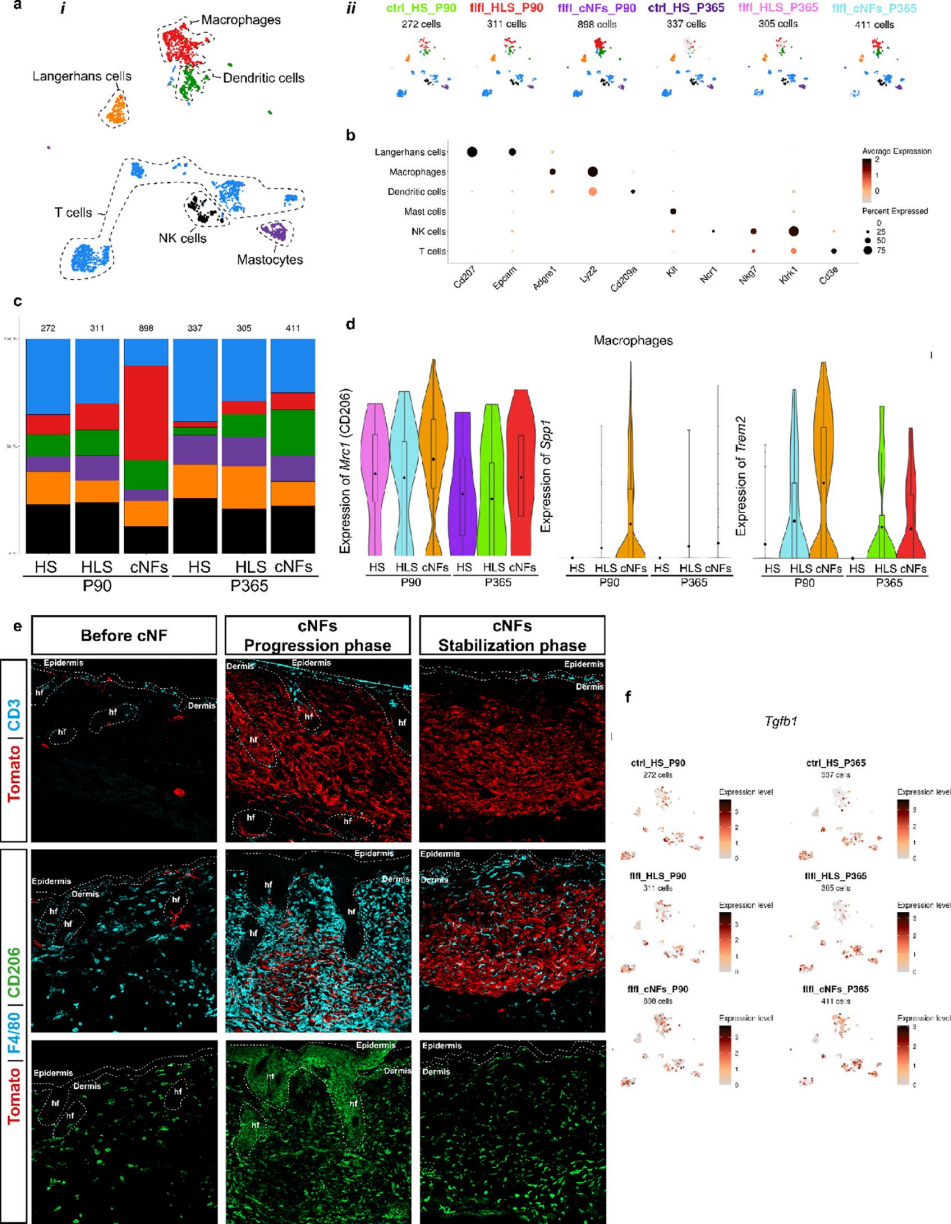
Immune landscape of cNFs. (**a*i***) Global UMAP plot of immune cells with their respective cell identities and (**a*ii***) UMAP plots separated by condition. (**b**) Dot plot showing the specificity of markers used to identify different immune cell types. (**c**) Distribution of the immune cell populations in the different conditions. (**d**) Violin plots showing the expression of *Mrc1* (CD206), *Spp1* and *Trem2* in macrophages in the different conditions. (**e**) Immunofluorescence showing Tom^+^ mutant SCs (Tomato^+^), wt T cells (CD3^+^), macrophages (F4/80^+^) and CD206^+^ macrophages (CD206+) from HLS skin, and cNFs, at the progression and stabilization phases. (**f**) Feature plot showing the expression of *Tgfb1* in immune cells in the different conditions. cNFs: cutaneous neurofibromas. HLS: healthy appearing skin. HS: healthy skin. P90: Postnatal Day 90. P365: Postnatal Day 365. SCs: Schwann cells

A small proportion of immune cell populations (300–900 cells per condition) were isolated, and their transcriptomic signatures were determined and compared with IHC profiling from our previous study [8]. Using a specific set of markers²², we identified macrophages, dendritic cells, Langerhans cells, T cells, NK cells, and mastocytes in all samples analyzed (Fig. 7a and b). Consistent with our previous findings [8], the proportion of macrophages was increased in growing cNFs compared to mature cNFs, HS, and HLS, suggesting a transient tumor infiltration. Further analysis of cNF-infiltrating macrophages revealed upregulation of *Mrc1*, which encodes the M2-like macrophage-associated CD206 protein (Figs. 7d–e). In addition, macrophages in growing cNFs exhibited increased expression of *Spp1* and *Trem2*, genes associated with organ fibrosis and tumorigenesis (Fig. 7d). Although *Tgfb1* was not differentially expressed, it was highly expressed in macrophages in all conditions (Fig. 7f).

Unexpectedly, T cells were the predominant immune cell type under all conditions in our data set (Fig. 7c). To further investigate their role in cNFs, we examined distribution of T cells in growing and mature cNFs using IHC and the CD3 marker (Fig. 7e). Despite their presence in the scRNAseq dataset, T cells were almost absent within cNFs themselves, localizing instead near hair follicles and the subepidermal border. This distribution suggests a minimal direct contribution of T cells to cNF development.

## Discussion

Despite the widespread occurrence of cNFs in almost all NF1 patients, their pathogenesis remains poorly characterized. A major limitation has been the lack of animal models that develop such benign tumors. In this study, we used a model that faithfully reproduces stepwise development of cNFs to construct a comprehensive transcriptomic atlas of tumor SCs and their microenvironment. Consistent with our previous study [8], we demonstrated that tumor SC proliferation is restricted to the growth phase. Furthermore, we showed that cNF growth is associated with increased profibrotic activity through the synergistic action of Fbs, tumor SCs, and pericytes, all of which orchestrate the synthesis of collagen and matrisome components. Finally, we discovered that tumor SCs express a set of genes that regulate axon growth and branching, likely contributing to the abnormally dense and disorganized innervation of cNFs. Collectively, our results highlight fibrosis and innervation as potential therapeutic targets, which suggests that targeting tumor SC proliferation in mature cNFs is unlikely to be effective.

We have developed a comprehensive single-cell transcriptomic atlas of control mouse skin, growing and mature cNFs, and their adjacent healthy-appearing skin. To optimize skin dissociation while preserving transcriptomic integrity, we compared short and long dissociation protocols. Although the long dissociation protocol yielded a higher proportion of non-keratinocyte cells and SCs, it significantly altered the transcriptomic signature of these cells. Therefore, we believe that by using a short dissociation procedure, our atlas accurately captures the transcriptomic activities of the major cell types in both control and mutant conditions. This dissociation time information should be carefully taken account of, when generating an unbiased transcriptomic atlas of cNFs from NF1 individuals.

Brosseau et al. previously performed a single-cell transcriptomic analysis of human cNFs [5]. However, several limitations of their study hinder direct comparison with our current atlas. First, their scRNA-seq data were derived exclusively from cNF tissue, without inclusion of adjacent healthy skin. Instead, they compared tumor-derived fibroblasts to previously published datasets from normal skin, raising concerns due to potential differences in dissociation protocols, patient backgrounds and anatomical sites. Moreover, their use of a prolonged overnight dissociation protocol may have altered the transcriptional profiles of cells, particularly non-tumoral cells like fibroblasts, as we have demonstrated in this study. Additionally, their dataset predominantly comprised fibroblasts, with limited representation of tumoral SCs, precluding analysis of their transcriptomic activity. Finally, while our study characterizes two distinct cNF developmental stages (progression and stabilization), their analysis likely focused on mature, stabilized tumors, as most cNFs in NF1 patients are quiescent with minimal MAPK pathway activity [9].

Our results point to the Aqp1^high^Nes^low^ SC population as the likely cell of origin for cNFs in our model. IHC validation revealed that Aqp1^high^Nes^low^ cells correspond to subepidermal glia located at the epidermis-dermis interface, where they envelop sensory nerve endings [1]. Their proximity to the skin surface and intimate contact with nerve endings, which are structures that are rich in molecular sensors for detecting various thermal and mechanical stimuli, suggest that such signals could serve as potential triggers for cNF tumorigenesis. Surprisingly, despite their initial location at the epidermis-dermis junction, the majority of cNFs in our model and in NF1 individuals are found in the deep dermis. Notably, we frequently observed a reduced density of Tom + SCs and innervation in the regions overlying cNFs in both mice and NF1 subjects. Accordingly, we hypothesize that “activated subepidermal glia” and their associated nerve endings could retract from their original position at the epidermis-dermis interface into the deeper dermis, potentially affecting the perception of thermal or mechanical stimuli at the tumor site. Monitoring subepidermal glia after localized skin trauma could provide further insight into this process.

A hallmark of the *Nf1* mutant Aqp1^high^Nes^low^ population is the activation of genes involved in axon growth, navigation, and branching during cNF development. We demonstrated that the innervation of both mouse and patient-derived cNFs is defasciculated, overly dense, and disorganized, which strongly suggests that tumor SCs contribute to this phenotype. In addition, we found that almost all tumor SCs remain in contact with the extra-numerical axons. While the function of this aberrant axonal network remains unclear, it is plausible that it helps tumor SCs maintain axon contact. This observation is in stark contrast with pNFs, where numerous SCs detach from axons and retain their proliferative activity [33]. Given that subepidermal glia engulf small-diameter nociceptive nerve endings [1], we propose that the abnormal innervation observed in cNFs corresponds to this type of sensory neuron. Interestingly, this neuronal population includes peptidergic neurons that secrete the neuropeptides CGRP or substance P, with well-established roles in cancer and other skin diseases [4, 7, 24, 26]. Further studies are needed to further elucidate the role of sensory neurons in cNF growth and maturation.

Additionally, using the *Prss56* NF1 mouse model, we observed that skin trauma promotes cNFs development, while Pan et al. [25] demonstrated that light exposure can drive optic glioma initiation in an NF1 mouse model. Interestingly, both triggers, skin trauma and light exposure, enhance neuronal activity, providing valuable insights into the role of neurons in NF1-associated tumors progression.

Another critical finding of our study is the increased profibrotic activity observed in the Aqp1^high^Nes^low^ SC population, pericytes, and dermal/epineurial Fbs. This activity is associated with the expression of a gene set encoding collagen and matrisome components that support the active synthesis and remodeling of the extracellular matrix. Most of these cells are quiescent or exhibit only transient proliferation, suggesting that fibrosis in this context occurs primarily within a quiescent cellular environment. In addition to the Aqp1^high^Nes^low^ SC population, dermal/epineurial Fbs and pericytes show increased expression of the *Tnc* and *Postn* genes. POSTN, whose expression is regulated by TGF-β in the skin, is a key contributor to ECM synthesis and assembly and plays a pivotal role in fibrosis pathogenesis through interactions with ECM components and integrin receptors [14, 22, 31, 34]. POSTN can also recruit M2 macrophages [2, 36, 38] and directly interact with sensory neurons to induce itching [19, 20]. Conversely, TNC, although not essential for ECM organization, is often upregulated in pathological conditions including wound healing, inflammation, tumorigenesis, and fibrosis [3, 18, 23, 35, 37]. TNC interacts with ECM components such as POSTN and cell surface receptors to influence cell proliferation, adhesion, and migration. It has also been implicated in abnormal nerve growth in the skin [6]. Given their diverse roles in fibrosis and related pathologies, TNC and POSTN are emerging as promising therapeutic targets for cNFs.

Finally, our study has several limitations. First, while the rapid sample dissociation protocol effectively preserves the transcriptomic signature, it reduces the total number of cells, potentially leading to the omission of rare immune cell populations. The use of single-nucleus RNA sequencing on the same sample types will help address this concern. In addition, all analyses were performed on mouse skin samples. Findings related to the pathological innervation of cNFs and tumor SC quiescence were subsequently validated using cNFs from patients. However, without performing transcriptomic analysis of patient tumors under optimal conditions, it remains challenging to determine the extent to which the transcriptomic signature of mouse cNFs accurately reflects that of patients.

## Electronic supplementary material

Below is the link to the electronic supplementary material.

Supplementary Material 1

## Data Availability

Single-cell RNA sequencing data are available as FASTQ files and raw count matrices in the Gene Expression Omnibus repository, under the accession number GSE289794. All scripts and complete methods used to process the raw count matrices to the figures are available via a Github repository (https://www.github.com/ppulh/cNFs_dvpt).

## Abbreviations

Adv-FIB: Adventitial fibroblasts
Anagen DP-FIB: Anagen dermal papillae fibroblasts
Aqp1: Aquaporin 1
BC: Boundary cap
BSA: Bovine serum albumin
cDNA: Cyclic DNA
cNF: Cutaneous neurofibromas
Dermal-FIB: Dermal fibroblasts
dpi: Days post-injury
DS-FIB: Dermal sheath fibroblast
ECM: Extracellular matrix
Endo-FIB: Endoneurial fibroblasts
Epi-FIB: Epineurial fibroblasts
FACS: Fluorescent activated cell sorting
Fb: Fibroblast
FBS: Fetal bovine serum
GEM: Genetically engineering model
HLS: Healthy-looking skin
HS: Healthy skin
Hyp-FIB: Hypodermal fibroblasts
IHC: Immunohistochemistry
MAPK: Mitogen-activated protein kinase
mSCs: Myelinating Schwann cells
Nes: Nestin
NF1: Neurofibromatosis type 1
Nf1-KO: Nf1 knockout
Nf1-KOi: cNF-inducible Nf1 knockout model
nmSCs: Non-myelinating Schwann cells
P45: Postnatal day 45
P90: Postnatal day 90
P365: Postnatal day 365
PBS: Phosphate-buffered saline
PCR: Polymerase chain reaction
Peri-FIB: Perineurial fibroblasts
pERK: Phospho-ERK
PFA: Paraformaldehyde
POSTN: Periostin
SC: Schwann cell
scRNAseq: Single-cell RNA sequencing
Telogen DP-FIB: Telogen dermal papillae fibroblasts
TGF-β: Transforming growth factor beta
TNC: Tenascin-C
Tom: Tomato

## References

[1] Abdo H, Calvo-Enrique L, Lopez JM, Song J, Zhang M-D, Usoskin D, El Manira A, Adameyko I, Hjerling-Leffler J, Ernfors P (2019) Specialized cutaneous Schwann cells initiate pain sensation. Science 365:695–699. 10.1126/science.aax6452 31416963 10.1126/science.aax6452

[2] Allard DE, Wang Y, Li JJ, Conley B, Xu EW, Sailer D, Kimpston C, Notini R, Smith C-J, Koseoglu E, Starmer J, Zeng XL, Howard JF, Hoke A, Scherer SS, Su MA (2018) Schwann cell–derived Periostin promotes autoimmune peripheral polyneuropathy via macrophage recruitment. J Clin Invest 128:4727–4741. 10.1172/JCI99308 30222134 10.1172/JCI99308 PMC6159985

[3] Bhattacharyya S, Wang W, Morales-Nebreda L, Feng G, Wu M, Zhou X, Lafyatis R, Lee J, Hinchcliff M, Feghali-Bostwick C, Lakota K, Budinger GRS, Raparia K, Tamaki Z, Varga J (2016) Tenascin-C drives persistence of organ fibrosis. Nat Commun 7:11703. 10.1038/ncomms11703 27256716 10.1038/ncomms11703 PMC4895803

[4] Blake KJ, Jiang XR, Chiu IM (2019) Neuronal regulation of immunity in the skin and lungs. Trends Neurosci 42:537–551. 10.1016/j.tins.2019.05.005 31213389 10.1016/j.tins.2019.05.005 PMC6661013

[5] Brosseau J-P, Sathe AA, Wang Y, Nguyen T, Glass DA, Xing C, Le LQ (2021) Human cutaneous neurofibroma matrisome revealed by single-cell RNA sequencing. Acta Neuropathol Commun 9:11. 10.1186/s40478-020-01103-4 33413690 10.1186/s40478-020-01103-4 PMC7792184

[6] Cai X, Han M, Lou F, Sun Y, Yin Q, Sun L, Wang Z, Li X, Zhou H, Xu Z, Wang H, Deng S, Zheng X, Zhang T, Li Q, Zhou B, Wang H (2023) Tenascin C + papillary fibroblasts facilitate neuro-immune interaction in a mouse model of psoriasis. Nat Commun 14:2004. 10.1038/s41467-023-37798-x 10.1038/s41467-023-37798-x PMC10086024 37037861

[7] Chamseddin BH, Hernandez L, Solorzano D, Vega J, Le LQ (2019) Robust surgical approach for cutaneous neurofibroma in neurofibromatosis type 1. JCI Insight 4:e128881. 10.1172/jci.insight.128881 10.1172/jci.insight.128881 PMC6629109 31038470

[8] Chéret J, Lebonvallet N, Carré J, Misery L, Le Gall-Ianotto C (2013) Role of neuropeptides, neurotrophins, and neurohormones in skin wound healing. Wound Repair Regeneration 21:772–788. 10.1111/wrr.12101 24134750 10.1111/wrr.12101

[9] Coulpier F, Pulh P, Oubrou L, Naudet J, Fertitta L, Gregoire J-M, Bocquet A, Schmitt A-M, Wolkenstein P, Radomska Kj, Topilko P (2023) Topical delivery of MEK inhibitor binimetinib prevents the development of cutaneous neurofibromas in neurofibromatosis type 1 mutant mice. Translational Res S1931524423001056. 10.1016/j.trsl.2023.06.003 10.1016/j.trsl.2023.06.003 37331503

[10] Di Tommaso P, Chatzou M, Floden EW, Barja PP, Palumbo E, Notredame C (2017) Nextflow enables reproducible computational workflows. Nat Biotechnol 35:316–319. 10.1038/nbt.3820 28398311 10.1038/nbt.3820

[11] Gresset A, Coulpier F, Gerschenfeld G, Jourdon A, Matesic G, Richard L, Vallat J-M, Charnay P, Topilko P (2015) Boundary caps give rise to neurogenic stem cells and terminal glia in the skin. Stem Cell Rep 5:278–290. 10.1016/j.stemcr.2015.06.005 10.1016/j.stemcr.2015.06.005 PMC4618659 26212662

[12] Hynes RO Naba A overview of the Matrisome—An Inventory of Extracellular Matrix Constituents and Functions 10.1101/cshperspect.a004903 PMC3249625 21937732

[13] Joost S, Annusver K, Jacob T, Sun X, Dalessandri T, Sivan U, Sequeira I, Sandberg R, Kasper M (2020) The molecular anatomy of mouse skin during hair growth and rest. Cell Stem Cell 26:441–457e7. 10.1016/j.stem.2020.01.012 32109378 10.1016/j.stem.2020.01.012

[14] Kalucka J, De Rooij LPMH, Goveia J, Rohlenova K, Dumas SJ, Meta E, Conchinha NV, Taverna F, Teuwen L-A, Veys K, García-Caballero M, Khan S, Geldhof V, Sokol L, Chen R, Treps L, Borri M, De Zeeuw P, Dubois C, Karakach TK, Falkenberg KD, Parys M, Yin X, Vinckier S, Du Y, Fenton RA, Schoonjans L, Dewerchin M, Eelen G, Thienpont B, Lin L, Bolund L, Li X, Luo Y, Carmeliet P (2020) Single-Cell transcriptome atlas of murine endothelial cells. Cell 180:764–779e20. 10.1016/j.cell.2020.01.015 32059779 10.1016/j.cell.2020.01.015

[15] Kii I (2019) Periostin functions as a scaffold for assembly of extracellular proteins. In: Kudo A (ed) Periostin. Springer Singapore, Singapore, pp 23–32 10.1007/978-981-13-6657-4_3 31037621

[16] Korsunsky I, Millard N, Fan J, Slowikowski K, Zhang F, Wei K, Baglaenko Y, Brenner M, Loh P, Raychaudhuri S (2019) Fast, sensitive and accurate integration of single-cell data with harmony. Nat Methods 16:1289–1296. 10.1038/s41592-019-0619-0 31740819 10.1038/s41592-019-0619-0 PMC6884693

[17] Kurtzer GM, Sochat V, Bauer MW (2017) Singularity: scientific containers for mobility of compute. PLoS ONE 12:e0177459. 10.1371/journal.pone.0177459 28494014 10.1371/journal.pone.0177459 PMC5426675

[18] Ly I, Romo CG, Gottesman S, Kelly KM, Kornacki D, York Z, Lee SY, Rhodes SD, Staedtke V, Steensma MR, Blakeley JO, Wolkenstein P (2023) Target product profile for cutaneous neurofibromas: clinical trials to prevent, arrest, or regress cutaneous neurofibromas. J Invest Dermatology 143:1388–1396. 10.1016/j.jid.2023.01.041 10.1016/j.jid.2023.01.041 37294242

[19] Mackie EJ, Halfter W, Liverani D (1988) Induction of Tenascin in healing wounds. J Cell Biol 107:2757–2767. 10.1083/jcb.107.6.2757 2462568 10.1083/jcb.107.6.2757 PMC2115631

[20] Mishra SK, Wheeler JJ, Pitake S, Ding H, Jiang C, Fukuyama T, Paps JS, Ralph P, Coyne J, Parkington M, DeBrecht J, Ehrhardt-Humbert LC, Cruse GP, Bäumer W, Ji R-R, Ko M-C, Olivry T (2020) Periostin activation of integrin receptors on sensory neurons induces allergic itch. Cell Rep 31:107472. 10.1016/j.celrep.2020.03.036 32268102 10.1016/j.celrep.2020.03.036 PMC9210348

[21] Nunomura S, Uta D, Kitajima I, Nanri Y, Matsuda K, Ejiri N, Kitajima M, Ikemitsu H, Koga M, Yamamoto S, Honda Y, Takedomi H, Andoh T, Conway SJ, Izuhara K (2023) Periostin activates distinct modules of inflammation and itching downstream of the type 2 inflammation pathway. Cell Rep 42:111933. 10.1016/j.celrep.2022.111933 36610396 10.1016/j.celrep.2022.111933 PMC11486451

[22] O’Dwyer DN, Moore BB (2017) The role of Periostin in lung fibrosis and airway remodeling. Cell Mol Life Sci 74:4305–4314. 10.1007/s00018-017-2649-z 28918442 10.1007/s00018-017-2649-z PMC5659879

[23] Ortonne N, Wolkenstein P, Blakeley JO, Korf B, Plotkin SR, Riccardi VM, Miller DC, Huson S, Peltonen J, Rosenberg A, Carroll SL, Verma SK, Mautner V, Upadhyaya M, Stemmer-Rachamimov A (2018) Cutaneous neurofibromas: current clinical and pathologic issues. Neurology 91. 10.1212/WNL.0000000000005792 10.1212/WNL.0000000000005792 29987130

[24] Ozanne J, Shek B, Stephen LA, Novak A, Milne E, Mclachlan G, Midwood KS, Farquharson C (2022) Tenascin-C is a driver of inflammation in the DSS model of colitis. Matrix Biology Plus 14:100112. 10.1016/j.mbplus.2022.100112 35669358 10.1016/j.mbplus.2022.100112 PMC9166467

[25] Pan Y, Hysinger JD, Barron T, Schindler NF, Cobb O, Guo X, Yalçın B, Anastasaki C, Mulinyawe SB, Ponnuswami A, Scheaffer S, Ma Y, Chang K-C, Xia X, Toonen JA, Lennon JJ, Gibson EM, Huguenard JR, Liau LM, Goldberg JL, Monje M, Gutmann DH (2021) NF1 mutation drives neuronal activity-dependent initiation of optic glioma. Nature 594:277–282. 10.1038/s41586-021-03580-6 34040258 10.1038/s41586-021-03580-6 PMC8346229

[26] Pinho-Ribeiro FA, Chiu IM (2019) Nociceptor nerves set the stage for skin immunity. Cell Res 29:877–878. 10.1038/s41422-019-0240-x 31619764 10.1038/s41422-019-0240-x PMC6889511

[27] Radomska KJ, Coulpier F, Gresset A, Schmitt A, Debbiche A, Lemoine S, Wolkenstein P, Vallat J-M, Charnay P, Topilko P (2019) Cellular origin, tumor progression, and pathogenic mechanisms of cutaneous neurofibromas revealed by mice with *Nf1* knockout in boundary cap cells. Cancer Discov 9:130–147. 10.1158/2159-8290.CD-18-0156 30348676 10.1158/2159-8290.CD-18-0156

[28] Rauen KA (2013) The rasopathies. Annu Rev Genom Hum Genet 14:355–369. 10.1146/annurev-genom-091212-153523 10.1146/annurev-genom-091212-153523 PMC4115674 23875798

[29] Sarin KY, Bradshaw M, O’Mara C, Shahryari J, Kincaid J, Kempers S, Tu JH, Dhawan S, DuBois J, Wilson D, Horwath P, De Souza MP, Powala C, Kochendoerfer GG, Plotkin SR, Webster GF, Le LQ (2024) Effect of NFX-179 MEK inhibitor on cutaneous neurofibromas in persons with neurofibromatosis type 1. Sci Adv 10:eadk4946. 10.1126/sciadv.adk4946 38691597 10.1126/sciadv.adk4946 PMC11062565

[30] Staedtke V, Topilko P, Le LQ, Grimes K, Largaespada DA, Cagan RL, Steensma MR, Stemmer-Rachamimov A, Blakeley JO, Rhodes SD, Ly I, Romo CG, Lee SY, Serra E (2023) Existing and developing preclinical models for neurofibromatosis type 1– Related cutaneous neurofibromas. J Invest Dermatology 143:1378–1387. 10.1016/j.jid.2023.01.042 10.1016/j.jid.2023.01.042 PMC11246562 37330719

[31] Stuart T, Butler A, Hoffman P, Hafemeister C, Papalexi E, Mauck WM, Hao Y, Stoeckius M, Smibert P, Satija R (2019) Comprehensive integration of Single-Cell data. Cell 177:1888–1902e21. 10.1016/j.cell.2019.05.031 31178118 10.1016/j.cell.2019.05.031 PMC6687398

[32] Varol C, Sagi I (2020) LOXL2 Inhibition paves the way for Macrophage-Mediated collagen degradation in liver fibrosis. Frontiers in Immunology 11 10.3389/fimmu.2020.00480 PMC7136575 32296422

[33] Wang Z, An J, Zhu D, Chen H, Lin A, Kang J, Liu W, Kang X (2022) Periostin: an emerging activator of multiple signaling pathways. J Cell Commun Signal 16:515–530. 10.1007/s12079-022-00674-2 35412260 10.1007/s12079-022-00674-2 PMC9733775

[34] Wolkenstein P (2001) La neurofibromatose 1. Med Sci (Paris) 17:1158–1167. 10.1051/medsci/200117111158

[35] Wu J, Williams JP, Rizvi TA, Kordich JJ, Witte D, Meijer D, Stemmer-Rachamimov AO, Cancelas JA, Ratner N (2008) Plexiform and dermal neurofibromas and pigmentation are caused by Nf1 loss in desert Hedgehog-Expressing cells. Cancer Cell 13:105–116. 10.1016/j.ccr.2007.12.027 18242511 10.1016/j.ccr.2007.12.027 PMC2846699

[36] Yamaguchi Y (2014) Periostin in skin tissue skin-Related diseases. Allergology Int 63:161–170. 10.2332/allergolint.13-RAI-0685 10.2332/allergolint.13-RAI-0685 28942960

[37] Yilmaz A, Loustau T, Salomé N, Poilil Surendran S, Li C, Tucker RP, Izzi V, Lamba R, Koch M, Orend G (2022) Advances on the roles of tenascin-C in cancer. J Cell Sci 135:jcs260244. 10.1242/jcs.260244 36102918 10.1242/jcs.260244 PMC9584351

[38] Zhou W, Ke SQ, Huang Z, Flavahan W, Fang X, Paul J, Wu L, Sloan AE, McLendon RE, Li X, Rich JN, Bao S (2015) Periostin secreted by glioblastoma stem cells recruits M2 tumour-associated macrophages and promotes malignant growth. Nat Cell Biol 17:170–182. 10.1038/ncb3090 25580734 10.1038/ncb3090 PMC4312504

