# Supplementary material for: Unraveling the development of cutaneous neurofibromas in neurofibromatosis type 1
Source: Acta Neuropathol Commun. 2025 Jul 19;13:158. doi: 10.1186/s40478-025-02075-z (PMC12275390; doi:10.1186/s40478-025-02075-z)
Supplement: Supplementary file 1 — Supplementary Material 1 [file 40478_2025_2075_MOESM1_ESM.docx]

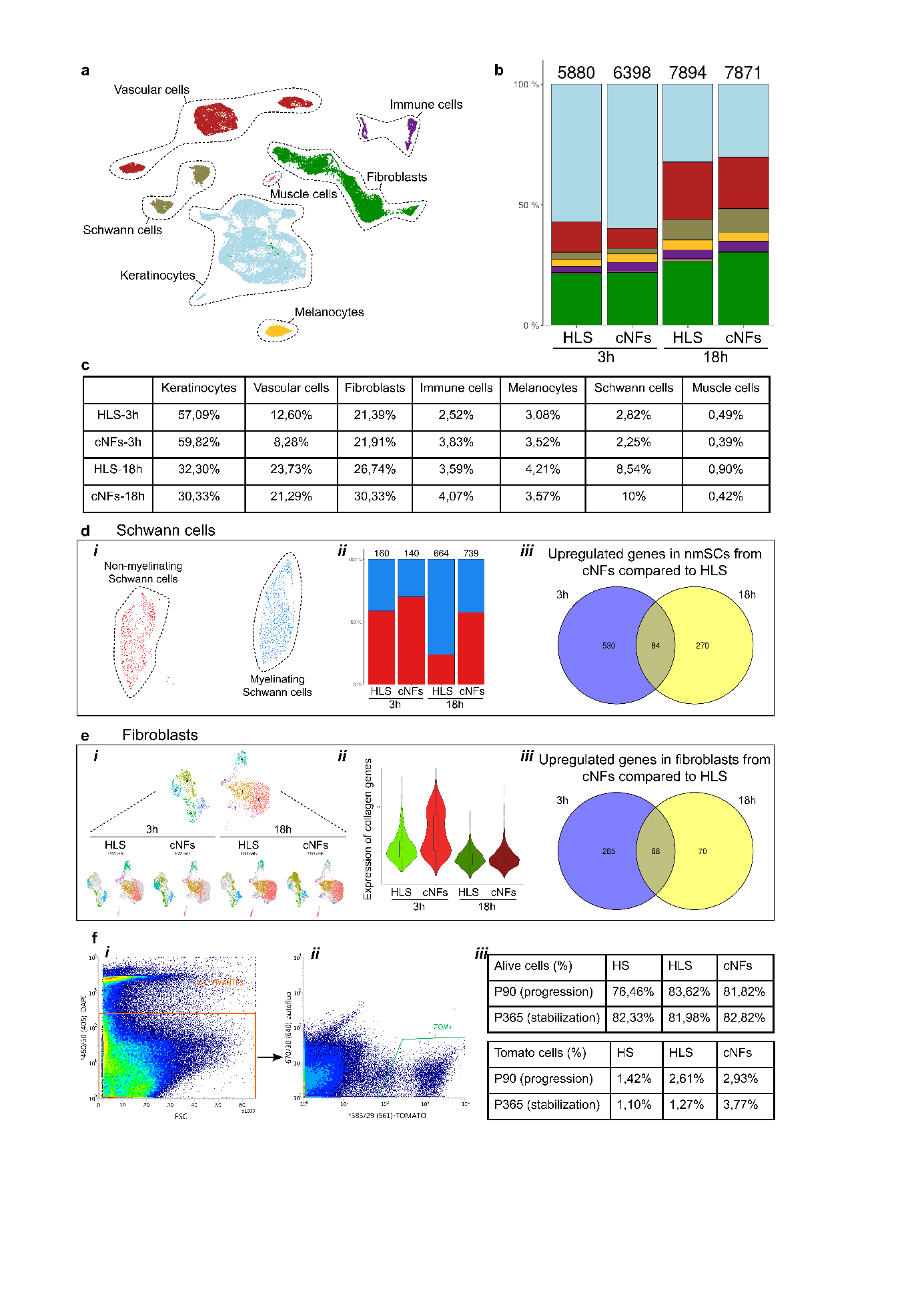


**Fig. S1 Comparison of the short and long dissociation protocols and FACS gating strategy.** (**a**) UMAPplot of cell types from dissociated skin. (**b**) Distribution of the skin cell populations in the different conditions and two dissociation protocols. (**c**) Proportions of the skin cell populations in the different conditions and two dissociation protocols. (**d*i***) UMAP plot of SCs from the whole skin samples. (**d*ii***) Distribution of the SC populations across the different conditions and dissociation protocols. (**d*iii***) Comparison of the number of upregulated genes in nmSCs from cNFs compared to HLS after the short (3h) or long (18h) dissociation protocols. (**e*i***) Global UMAP plot of fibroblasts from the whole skin samples and UMAP plots separated by condition and dissociation protocol. (**e*ii***) Violin plot showing global collagen gene expression in fibroblasts across the different conditions and dissociation protocols. (**e*iii***) Comparison of the number of upregulated genes in fibroblasts from cNFs versus HLS after the short (3h) or long (18h) dissociation protocols. (**f*i*-*ii***) Representative data from FACS sorting of freshly isolated total mouse skin cells. After gating out DAPI- cells, Tom+ freshly isolated mouse skin cells were sorted for scRNAseq. (**f*iii***) Proportions of alive cells (DAPI-) and Tom+ cells in freshly isolated mouse skin cellpreparations. cNFs: cutaneous neurofibromas. HLS: healthy appearing skin. HS: healthy skin. P90: Postnatal Day 90. P365: Postnatal Day 365.


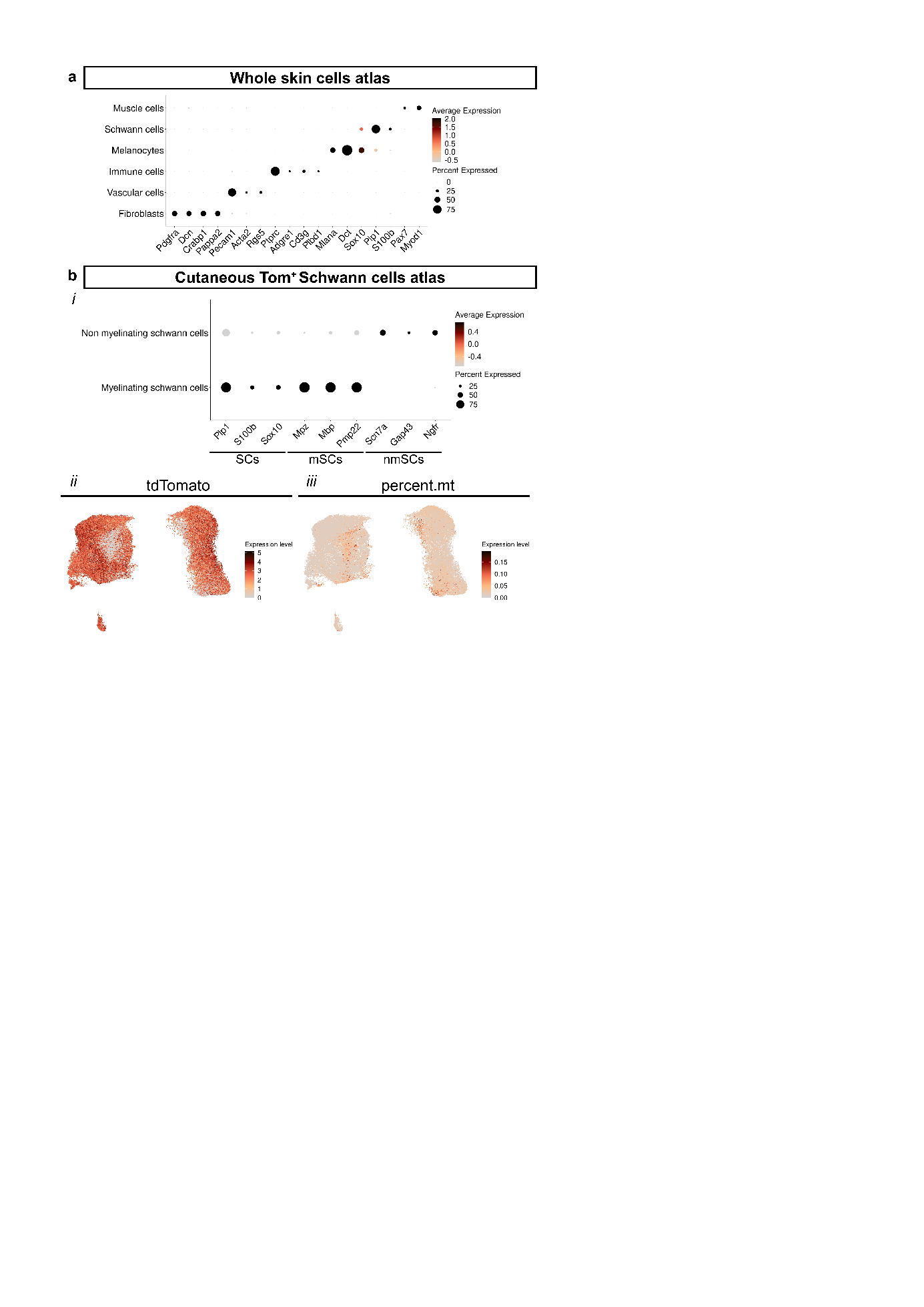


**Fig S2 Cluster identities in whole skin cell and cutaneous Tom+ SC atlases.** (**a**) Dot plot illustrating the specificity of markers used to assign cell identities in the whole skin cell atlas. (**b*i***) Dot plot illustrating the specificity of markers used to differentiate between myelinating and non-myelinating Tom+ Schwann cells (SCs). (**b*ii-iii***) Feature plots illustrating the expression of tdTomato and mitochondrial genes within the cutaneous Tom+ SC atlas. SC: Schwann cell.


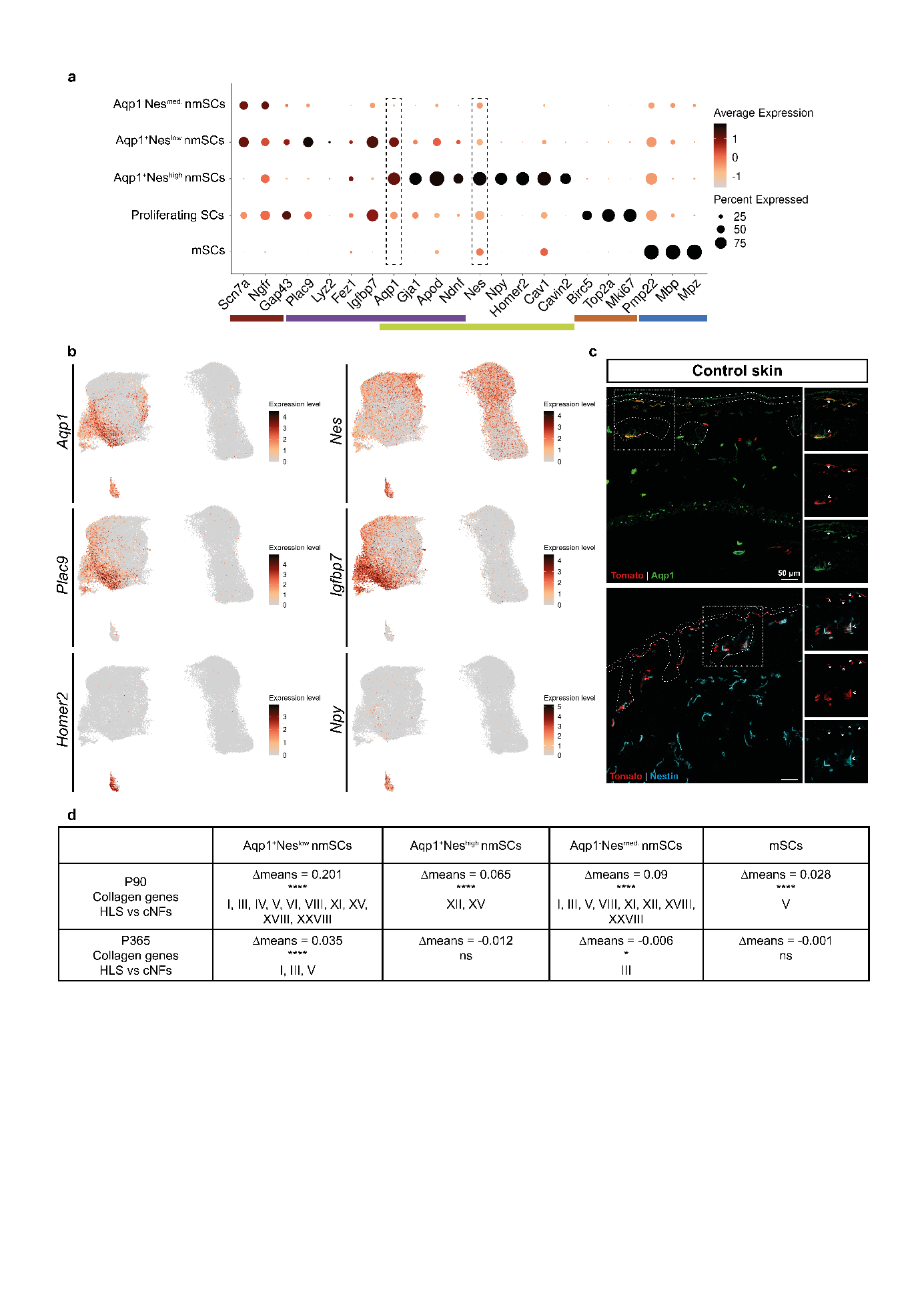


**Fig. S3 Mapping the identity and location of SC subpopulations and their collagen production**. (**a**) Dot plot showing the specificity of selected markers used to define subidentities within the cutaneous Tom+ SC dataset. (**b**) Feature plots showing the expression patterns of specific markers across the dataset. (**c**) Immunofluorescence images of control skin at P45 showing Tom+ tracked SCs expressing aquaporin-1 (Aqp1^+^) and nestin (Nestin^+^). (**d**) Table summarizing differences in global collagen gene expression between cNFs and HLS across different stages and SC subpopulations. cNFs: cutaneous neurofibromas. HLS: healthy appearing skin. P45: Postnatal Day 45. SC: Schwann cell.


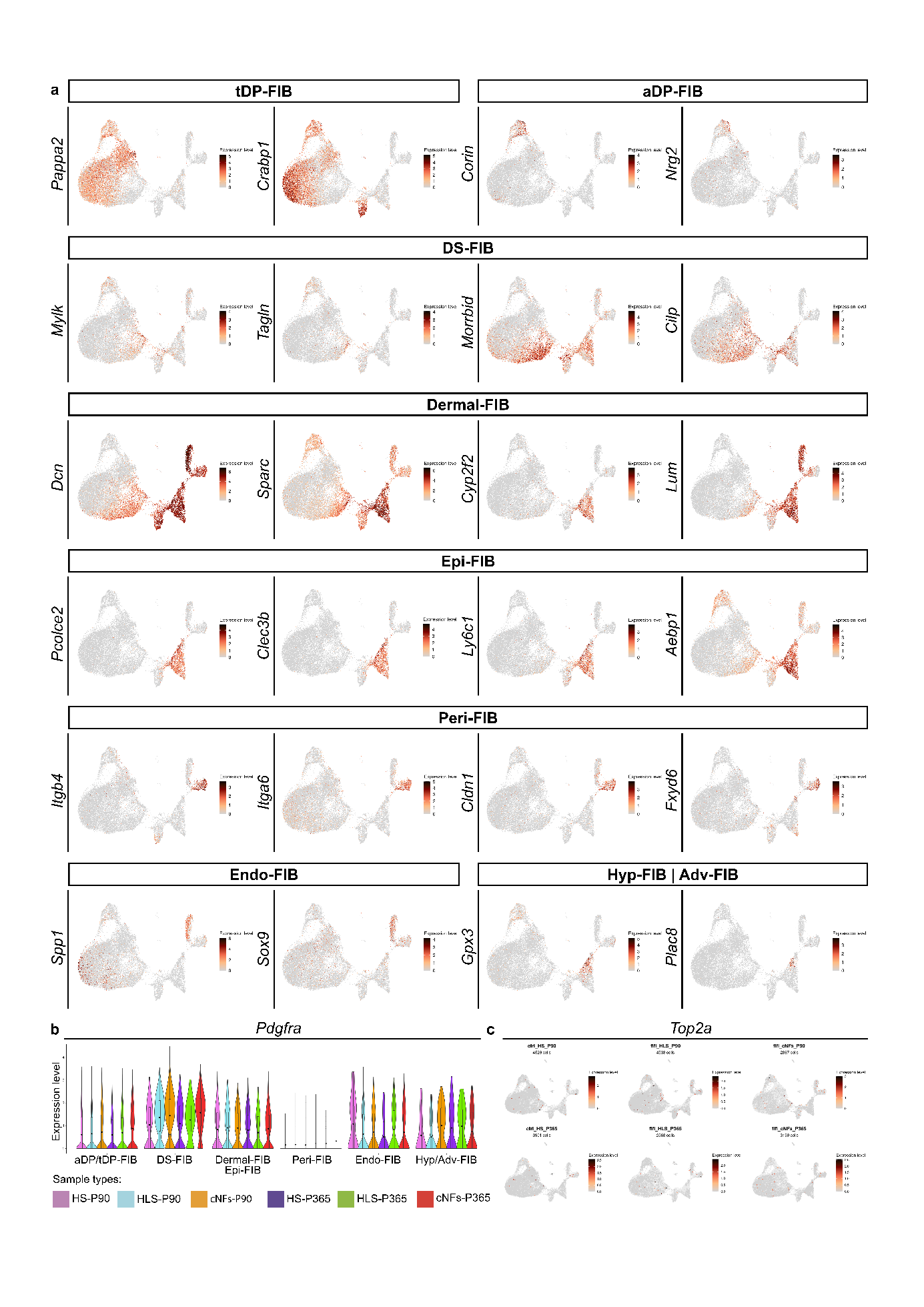


**Fig. S4 Profiling of fibroblast subpopulations from cNFs**. (**a**) Feature plots illustrating the specificity of selected markers used to define subpopulations within the fibroblast dataset.
(**b**) Violin plot showing ***Pdgfra*** gene expression across different conditions and fibroblast subpopulations. (**c**) Feature plot showing the expression of ***Top2a***, a marker of dividing cells, across different conditions in fibroblast populations. aDP/tDP-FIB: anagen and telogen dermal papilla fibroblasts. DS-FIB: dermal sheath fibroblasts. Hyp/Adv-FIB: hypodermal/adventitial fibroblasts. Endo-FIB: endoneurial fibroblasts. Peri-FIB: perineurial fibroblasts. Dermal-FIB | Epi-FIB: dermal and epineurial fibroblasts. cNFs: cutaneous neurofibromas. HLS: healthy appearing skin. HS: healthy skin. P90: Postnatal Day 90. P365: Postnatal Day 365.

**
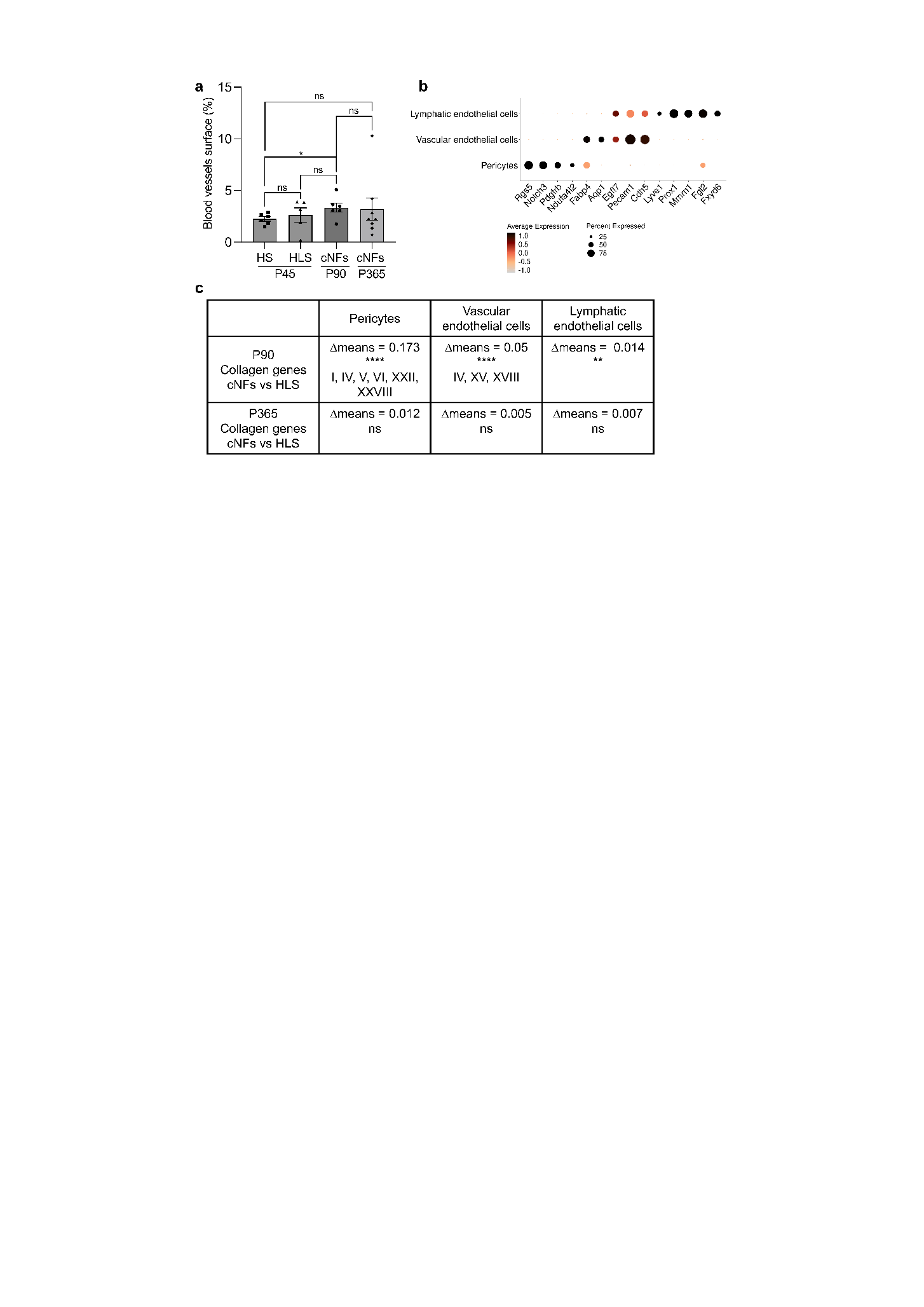
**

**Fig. S5 Profiling of vascular cells from cNFs.** (**a**) Quantification of the PECAM^+^ staining area per field across conditions. (**b**) Dot plot showing the specificity of markers used to assign vascular cell identities. (**c**) Table summarizing the differences in global expression of collagen genes between the cNFs and HLS across the different conditions and vascular cell subpopulations. cNFs: cutaneous neurofibromas. HLS: healthy appearing skin. HS: healthy skin. P45: Post-natal day 45. P90: Postnatal Day 90. P365: Postnatal Day 365.
